# Inversion symmetry and local vs. dispersive interactions in the nucleation of hydrogen bonded cyclic n-mer and tape of imidazolecarboxamidines

**DOI:** 10.3762/bjoc.4.23

**Published:** 2008-07-07

**Authors:** Sihui Long, Venkatraj Muthusamy, Peter G Willis, Sean Parkin, Arthur Cammers

**Affiliations:** 1 University of Kentucky, Department of Chemistry, Lexington, KY. 40506-0055

**Keywords:** counterpoise, crystal engineering, packing motif, solution conformation, Wallach's rule

## Abstract

Substitutional changes to imidazolecarboxamidine that preserved intermolecular hydrogen bonding in the solid state were used to study the relationship between packing and the hydrogen bond motif. Various motifs competed, but the most common imidazolecarboxamidine crystalline phase was a *C*_i_ symmetric dimer that established inversion centers by associating enantiomeric tautomers. Counter to intuition, the calculated gas-phase energies per molecule of the solid state atomic coordinates of the *C*_i_ dimer motifs were higher than those of the *C*_1_ dimer, trimer, tetramer and tape motifs, while the packing densities of *C*_i_ dimers were found to be higher. This result was interpreted as an enhanced ability of the *C*_i_ dimers to pack. If other motifs competed, the hydrogen bonds and conformations should be lower in energy than the *C*_i_ dimer. The results detail the effect of packing on the conformation in these molecules. The results are interpreted as a rough measure of the energetic compromise between packing and the energies related to the coordinates involving one dihedral angle and hydrogen bonding. The results establish a connection between solution and solid phase conformation.

## Introduction

Bonding in organic compounds and nuances inherent in crystal packing engender boundless diversity in the arrangements of hydrogen bonded organic solid states. Due to its relative strength and its directional nature, the hydrogen bond has drawn much attention as a structural element in the design of crystalline phases [1–5]. The paradox that hydrogen bonding is important as both an element of structural diversity and design vanishes given that diversity depends on maximizing the number of hydrogen bonding options available to molecules whereas design focuses on controlling the direction and minimizing the number of hydrogen bonds. The current approach to probing relationships between molecular structure and packing involves substitutional modifications to an interesting parent molecule with limitations on hydrogen bond structural diversity [6–8].

**Figure 1:**
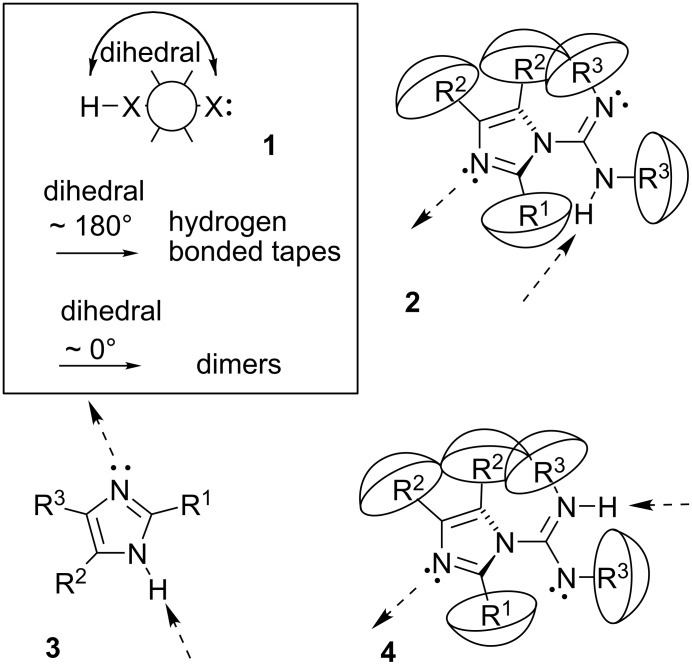
**1:** An intuitive prediction regarding the relationship between crude hydrogen bond donor/acceptor directionality and hydrogen bond molecularity. **2:** The amidine and imidazole moieties are not coplanar due to steric interactions; the CN–CN dihedral angle (50–90°) directs hydrogen bond vectors (dashed arrows). **3:** Imidazole prefers the tape. **4:** This tautomer/rotamer is not observed.

Hydrogen bonds optimally positioned, **1** (Figure 1), can lead to infinite polymers (tape) [9–10], cyclic n-mers [2,11–12], or dimers. For example, imidazolecarboxamidines **2** and imidazoles **3** have similar hydrogen bonding options offering sp^2^-NH hydrogen bond donors and sp^2^-N atom hydrogen bond acceptors. However **3** directs the hydrogen bond donor and acceptor approximately linearly [13] versus the ~90° intramolecular dihedral angle in **2**. Large angles between hydrogen bond donor and acceptor, as in **3**, should predispose solid state tapes [13], whereas small angles should prefer dimers. Planar motifs allow the favorable linearity in the hydrogen bond angles [14–16]. Intermediate dihedral angles ought to straddle the two crystalline phase motifs in the production of rings larger than dimers. The hydrogen bond motifs of amides encapsulate this concept in that tape and dimer dominate the solid state [3,8,17]. Crystalline phase hydrogen bonding near the parametric tipping point [18] between 0° and 180° might likely afford a variety of hydrogen bonded motifs.

To simplify the interplay between directionality and the motif of the crystalline phase n-mer we studied a molecule with one hydrogen bond donor and one acceptor. Motif diversity increases sharply with more donors or acceptors [2]. The intermolecular hydrogen bond between the amidine sp^2^-NH and the imidazole sp^2^-N was maintained in all crystalline phases examined. Apparently the sp^2^-N atom in the imidazole accepts hydrogen bonds better than the sp^2^-N atom in the amidine group, and the non-involvement of amidine, as the weaker electron donor, agrees with previous studies of competitive solid state hydrogen bonding [19]. Rotamer/tautomer **4** was not observed in the crystalline phases, providing further control and predictability.

Crystallization requires non-equilibrium conditions to progress [20–21]; however, predictions are usually modeled based on thermodynamic considerations. With the notion that packing enthalpy mandates the crystalline phase [22], the question asked by this study was: how do the stabilities of the hydrogen bonded n-mers compare energetically in the absence of packing?

## Results and Discussion

**Figure 2:**
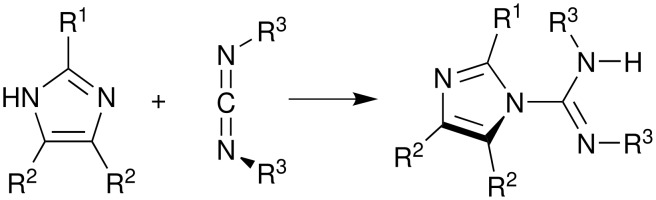
Facile syntheses of imidazole carboxamidines from commercial imidazoles and carbodiimides furnished a series of crystalline phases with related hydrogen bonding.

The molecules in Table 1 were synthesized by combining imidazoles with commercially available carbodiimides as in Figure 2. Even though the synthesis is easy, these molecules are very rare in the chemical literature. The products were crystallized under various conditions. In one of 21 syntheses, (R^1^ = NH_2_) a more complex molecule than the carboxamidine was isolated due to the inclusion of two carbodiimide moieties in the product; see Supporting Information File 1. Two other crystalline phases, with R^1^ = NH_2_, favored hydrogen bond tapes with the involvement of R^1 ^hydrogen bonding. We spent little time on these structures; they are not included in the current study. However, the preparations of these are included in the Supporting Information File 1. Hydrogen bonding in the remaining substances was categorized into four groups: cyclic *C*_i_ dimers, cyclic *C*_1_ dimers, cyclic n-mers and infinite tape. Without the addition of a hydrogen bond donor for R^1^, the tape motif appears to be unlikely. Some effort was made to find polymorphs. Vials of material were arrayed in a variety of solvents and the unit cells were indexed. The same solvent-free crystal structures or crystals not suitable for diffraction (disordered or too small) were obtained.

**Table 1:** The imidazolecarboxamidines synthesized and crystallized for this study. Given are the hydrogen bond molecularity, the imidazole/amidine dihedral angle (θ), and the space group. Further information regarding data collection can be found in the Supporting Information File 2.

Amidine ──────────── Imidazole		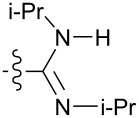 **a**	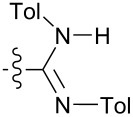 **b**	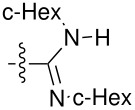 **c**

**5**	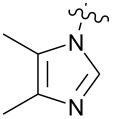	no crystal	dimer ±81.5°, *P*-1	dimer ±87.0°, *P*2_1_/c hexane or EtOAc
**6**	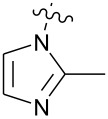	no crystal	trimer −102.6°, 108.3°, (104.3°, −98.6°), *P*2_1_/c	dimer ±66.7°, *C*2/c hexane or EtOAc
**7**	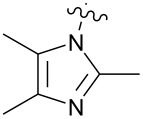	dimer ±91.8°, *P*-1 hexane	dimer ±100.7°, *P*2_1_/n MeOH/EtOAc	(1) dimer ±108.5°, *P*2_1_/n, hexane ------------ (2) dimer polymorph −91.8°, 91.0°, *P*2_1_/c
**8**	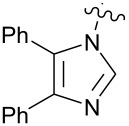	*C*_1_ dimer 56.8°, 67.8°, *P*-1 hexane or EtOAc	*C*_1_ dimer 95.5°, 60.5°, *P*2_1_/n	no crystal
**9**	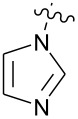	tetramer 101.2°, −100.1°, 103.0°, −106.0°, *P*2_1_/n hexane	(1)−(3) dimer ±54.3°, *P*2_1_/n ±53.0°, *P*2_1_/n ±54.1°, *P*2_1_/n (1) EtOAc, (2) C_6_H_5_CH_3_, (3) Et_2_O all 1:1	dimer ±88.0°, *P*2_1_/n
**10**	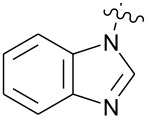	no crystal	dimer ±91.1°, *P*-1	dimer ±73.3°, *P*-1 hexane or EtOAc
**11**	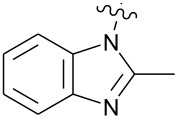	tape 63.2°, *P*2_1_/c	dimer ±99.7°, *P*2_1_/n	dimer ±98.7°, *P*-1

If not noted, crystals were from EtOAc.

Hydrogen bonded dimers possessing inversion centers, *C*_i_ dimers, comprised the most popular bonding motif found in the solid state imidazolecarboxamidines. To investigate the nature of this energetic preference we compared by computation the gas-phase stabilities of these dimers versus the other observed hydrogen bonded motifs. Counter to intuition, in the absence of packing interactions the *C*_i_ dimers in their crystalline phase atomic coordinates were calculated to be considerably less stable than the less popular structural motifs.

Literature on organic solid states contains much dialog regarding the minimization of Z' (molecules in the asymmetric unit) by associating structural or conformational enantiomers. Wallach's hypothesis foreshadowed this dialog: the racemic crystalline phase is more dense and more stable than the analogous optically pure crystalline phase [23]; although, exceptions have been noted [24]. Musing about this issue, Brock and Dunitz state, “Inversion centers are especially favorable for crystal packing because they diminish like-like interactions and are uniquely compatible with translation.” [25] Symmetry is a powerful component in packing: 83% of the entries in the Cambridge Structural Database that do not symmetry-relate molecules possess pseudosymmetry within 0.5 Å [26]. Molecules capable of either chiral or achiral space groups prefer the latter with concomitant minimization of Z'. The infrequency of chiral space groups in the CSD (~1:9) [27] may manifest a bias in the data toward small Z' possessing inversion symmetry.

Packing could select a particular motif because dispersion forces factor in the construction of the organic solid state [28–29]. If modern incantations of Wallach's hypothesis apply, the preferred solid state motif of **2** is likely the *C*_i_ dimer and structures that successfully compete with the *C*_i_ dimer should have increased stabilities from identifiable atomic parameters.

**Figure 3:**
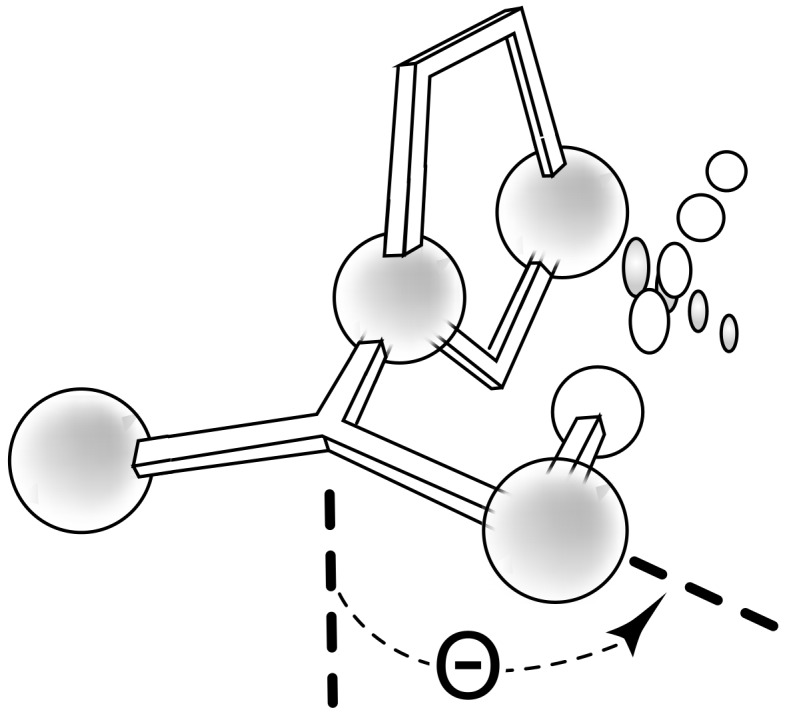
The NCNC dihedral angle, θ, between the hydrogen bond donors and acceptors, was assigned values between +180° and −180°. Structures with opposite signs are conformational enantiomers.

The dihedral angles, θ, between the imidazole and the amidine moieties characterize the solid-state conformation and are reported in Table 1. This parameter is defined in Figure 3 by the amidine N, C atoms and imidazole N, C atoms. With all else equal, molecules with θ of equal value but of opposite sign are conformational enantiomers.

Even though only five structures did *not* crystallize as hydrogen bonded *C*_i_ dimers, comparing the atomic parameters of these to the atomic parameters of the *C*_i_ dimers is instructive. The directionality of the hydrogen bonds in this family of molecules approximates the hypothetical tipping point between atomic parameters that favor the infinite hydrogen bonded tape motif and the dimer. Despite the fact that the *C*_i_ dimer was the most common, the *C*_1_ dimer, trimer, tetramer and tape are calculated below to have more stable hydrogen bonding. The *C*_i_ dimer also tended to have the calculated least stable θ dihedral angles. Compensative packing must render the *C*_i_ dimer competitive.

**Figure 4:**
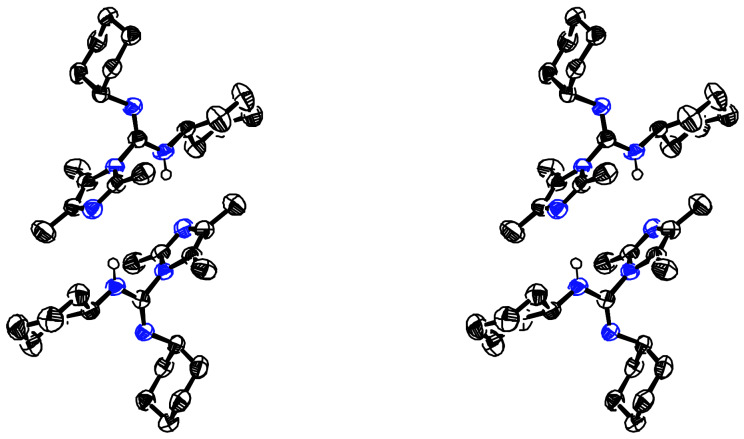
Stereoview of Dimer **5c**. This dimer stacked imidazole rings with R^1^ pointing in opposite directions.

Cursory examination of molecular models shows that the imidazole moieties could stack with the R^1^ substituents pointing either in the same or, as in structure **5c** in Figure 4, in opposite directions. R^1^ substituents pointing in opposite directions were the most popular, occurring in 12 of the 14 dimers. In light of Wallach's rule, an obvious advantage of this arrangement is the possibility that the dimeric units possess an inversion center and afford the molecules the assumed advantage of pairing two conformational enantiomers. The molecules that crystallized as dimers of conformational enantiomers with Z' = 1 had +/− pairs of single valued θ that varied between absolute values of 54° and 114°.

The only polymorph found in this study, dimer **7c2**, nearly missed the inversion center (root mean square difference from a perfect inversion center of the C and N atoms = 0.03 Å, θ = 91.0°, θ' = −91.8°, Z' = 2). This is a common situation [26]. For perspective on this result, *C*_1_ dimers **8a** and **8b** missed the inversion center by RMS differences ~8.0 Å, a distance similar to the dimensions of the unit cell. The space groups encountered in this study were without exception achiral so the dimers with θ values: 91.8 and −91.0 were also present in the crystalline phase of **7c2**. Due to its structural proximity to *C*_i_, for the purpose of taxonomy, **7c2** was classed as a *C*_i_ dimer. Differences between **7c2** and polymorph **7c1**, a true *C*_i_ dimer, are discussed below.

When the dimer crystallized with the two R^1^ substituents pointing in the same direction, θ and θ' within the dimer had different values of the same sign. Nature did not use a *C*_2_ operation to symmetrize these values. Only two molecules, **8a** and **8b** crystallized as *C*_1_ dimers, thus limiting any generalizations about the range of θ in these cases. The *C*_1_ dimers paired their aromatic substituents at R^2^ in **8a** and **8b** and at R^3^ in **8b**. Optimizing π-stacking, hydrogen bonding and θ likely allowed these two *C*_1_ dimer solid states in lieu of the otherwise ubiquitous *C*_i_ dimer.

**Figure 5:**
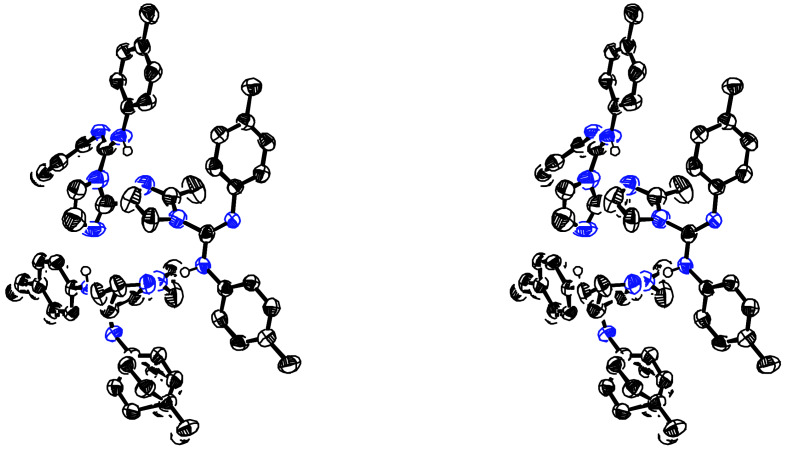
Stereoview of trimer **6b**.

There were three other imidazolecarboxamidines in this study that did not crystallize as dimers: trimer **6b**, tetramer **9a** and tape **11a** (see Table 1 and Figure 5–Figure 7). Like the *C*_1_ dimers **8a** and **8b**, structures **6b**, **9a** and **11a** tended to possess more stable calculated θ angles and hydrogen bonds than those found in the *C*_i_ dimers. Structures **6b** and **9a** are interesting in their putative ontological relationship to the dimers. One molecule in the trimer asymmetric unit was disordered. The two ordered molecules were analogous to an open *C*_i_ dimer with θ angles numerically close but of opposite sign. The best solution of the disorder modeled two molecules with large θ of opposite signs with unequal levels of occupancy. Tetramer **9a** does not suffer from this ambiguity; it is approximately an open dimer of *C*_i_ dimers with large θ of alternating sign. The molecules in the trimer and tetramer are unrelated by symmetry; Z' = 3 and 4 respectively.

**Figure 6:**
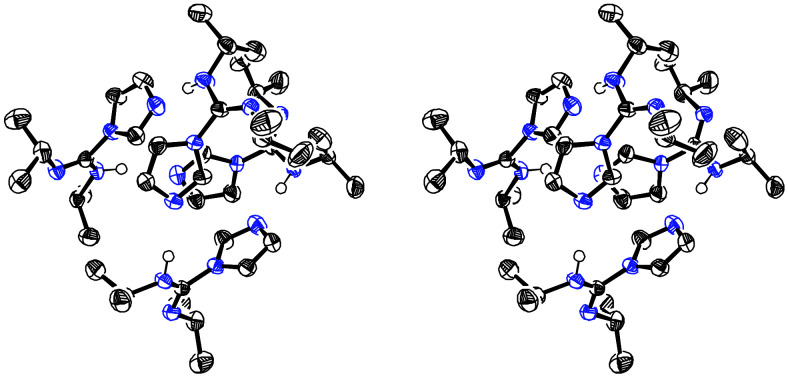
Stereoview of tetramer **9a**.

**Figure 7:**
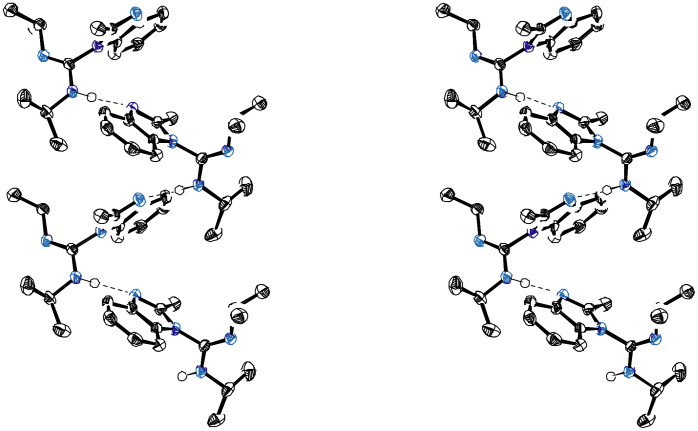
Stereoview of linear hydrogen bond tape **11a**.

A comparison of the calculated stabilities of the sets of hydrogen-bound n-mers to determine if any hydrogen bonded motif enjoyed an energetic advantage follows in the text below. Two approaches based on the principle of practical model chemistries [30] queried the stabilities of the crystalline phase n-mers in the absence of packing.

**Figure 8:**
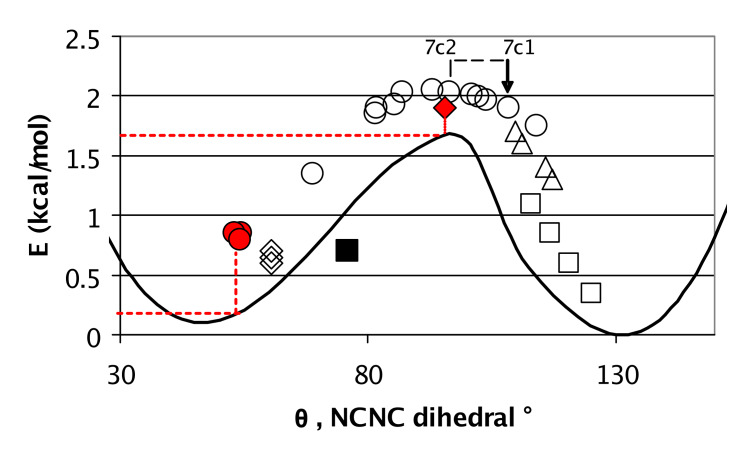
The calculated (rhf/6-311+g(d,p)) potential energy (kcal/mol) of *N,N*'-dimethyl-1H-imidazole-1-carboxamidine, **2**, R^1^,R^2^=H, R^3^=Me versus dihedral angle θ (degrees). Icons represent crystal structure θ values: (∘) = *C*_i_ dimer, (◊) = *C*_1_ dimer, (□) = trimer, (▵) = tetramer, (■) = tape. The red icons are in apparent contradiction of the trend: the *C*_2_ dimer has high-energy θ; these are discussed further.

The potential energy of the imidazolecarboxamidine as a function of θ (Figure 8) was investigated by performing relaxed scans with *Gaussian (G03)* [31] at rhf/6-311+g(d,p) on hypothetical model monomer **2a** (R^1^, R^2^ = H and R^3^ = CH_3_). Figure 8 plots the potential energy of **2a** as a function of θ. The experimental crystal structure θ parameters of the molecules in Table 1, categorized by hydrogen bond motif are included on the graph. At θ angles near 0° or 180° steric factors should increase the energies associated with θ and bring into the question whether using **2** as a model for the θ energy in all structures is useful. However, from θ = 50–130° a variety of steric environments are present in **5**–**11**. For example the steric nature of Table 1 entries **11** and **7** could possibly constrain θ to ~90°, but they do not; θ for **7** is near 90°, but θ for **11** is relatively small. Independent of sterics, the *C*_i_ dimers on average clustered in the high-energy area around θ ~90–100° in Figure 8 whereas the other crystalline phases tended to have θ parameters associated with lower energies corresponding to the gains in π bonding as θ approached planarity. The dashed arrow shows how the energetic content associated with θ of **7c2** changed when switched to the true *C*_i_ polymorph, **7c1**. The red icons represent *C*_i_ dimers with θ of exceptional stability and a parameter in a *C*_1_ dimer that is relatively unstable; these are discussed below.

A more holistic calculation that allowed gross comparisons of the stabilities of all solid-state n-mers in the absence of packing interactions yielded paradoxical conclusions similar to the preceding calculations presented in Figure 8. These calculations are more holistic in that more than one parameter is the focus of the calculation and the results are paradoxical because the more popular motif, the *C*_i_ dimer, is again calculated to be less stable.

**Figure 9:**
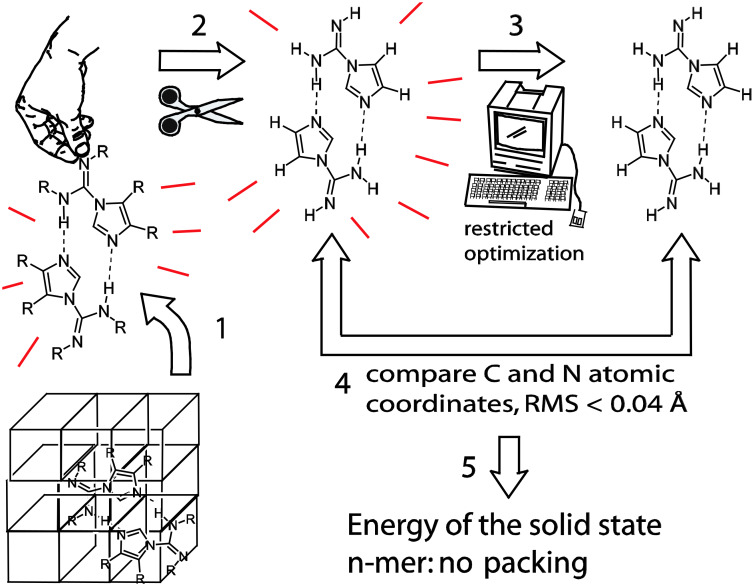
A Flow chart for the calculation of the energies of the n-mers minus the effects of packing and substituent interactions. See text.

The steps of this calculation are a bit complex; a flow chart is presented in Figure 9. Step 1: crystallographic information files (cif) were written as *Gaussian (G03)* input files, thus removing the material from the crystalline phase and bringing it into the gas-phase. Step 2: The atoms corresponding to R^1^, R^2^ and R^3^ were replaced with hydrogen atoms while preserving the relative positions of the remaining heavy atoms, this gives a set of structures corresponding to **2b**, R^1^, R^2^, R^3^, = H, that differ only in hydrogen bonded motif and atomic position. For the size of the molecules under study, accurately calculating dispersion forces in the clusters would have entailed an unreasonable high level of theory [32]. Step 3: The NHN hydrogen bond lengths, dihedral angle θ, four inter-imidazole-ring bond angles and one Cartesian coordinate per molecule were frozen. The remaining atomic parameters were optimized at the rhf/6-311+g(d,p) level of theory. It is important to optimize the C-H and N-H bond lengths to remove crystallographic errors generated by the algorithmic assignment of H atom positions. Step 4: Assurance that the solid state coordinates were not severely perturbed by optimization was gained from C- and N-atom RMS differences between the X-ray structure coordinates and those of the corresponding optimized structures; the RMS differences were calculated using gOpenMol. An RMS difference value of 0.04 Å was tolerated (0.02 Å average RMS difference). The two high values near 0.035 Å were not consequential. Step 5: Basis set superposition error (BSSE) biases the calculation of the hydrogen bond energies [33–34]; therefore, counterpoise correction was applied. The per-molecule, mostly-strong-local-energetic contributions to the stabilities of the n-mers were accessible by simply dividing the energies from these calculations by n. This calculation should include the effects of θ and hydrogen bonding.

Calculating the analogous stability of the linear hydrogen bonded tape, **11a**, was more complicated. The corresponding linear dimer, trimer, tetramer and pentamer of **11a** were subjected to the above method. From the slope of the energy/n vs. n relationship, the per-molecule energy of the linear hydrogen bonded tape was calculated. The effect of the non-hydrogen bonded termini was further diminished by extrapolating the curve to n = 100.

**Figure 10:**
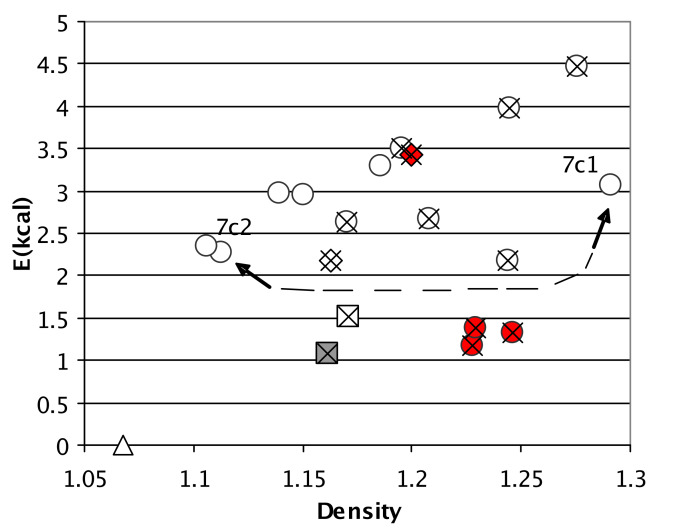
Icons correspond to those in Figure 8. Crosses indicate structures with aromatic groups. The calculated (rhf/6-311+g(d,p)) energy (kcal/mol) with counterpoise BSSE correction of **2b** (Figure 1: R^1^−R^3^ = H) in the particular crystal structure atomic coordinates versus empirical packing density. Broad conclusions: The crystalline phases with aromatic groups were denser. Gas phase hydrogen bonds in the *C*_i_ dimers were less stable but *C*_i_ solid states were denser; the red icons are obvious exceptions. See text.

Since all values of n-mer/n are associated with the same molecular formula **2b**, the energies per molecule allow fair comparison of the energies due to the pi-energy effect of θ and hydrogen bonding in the n-mers in the absence of packing. Figure 10 summarizes the results of 20 calculations of this type by graphing the calculated, gas-phase, n-mer/n energies against the packing densities (molecular mass ⋉ Z/cell volume).

With all else equal, packing density should correlate only grossly with solid state intermolecular interactions [22,24,26,35]. Stark differences in hydrogen bonding should enhance this correlation because the strength and directionality of hydrogen bonding can perturb packing in organic solids. Kitaigorodskii posits that organic molecules in crystalline phases fill space nearly as efficiently as close-packed spheres ~0.74 [35]. This occurs when the dimples and bumps of one molecule spatially correspond with the bumps and dimples of a lattice mate. Structures reliant on hydrogen bonds could violate this general rule by decreasing the packing coefficient due to the directionality of hydrogen bonds [36]. Figure 10 shows that the structures with aromatic substituents (X's in the graph) tended to be denser. In general, aromatic organics are denser than aliphatic organics [37]. This is likely due to the fact that bonds are shorter for sp^2^/sp^2^ atoms than for sp^3^/sp^3^ atoms. The effect of aromaticity on density is likely enhanced because these double aromatic substituents in these small structures accounted for much of the molecular mass.

In Figure 10, calculations again find that the *C*_i_ dimers (frozen crystalline phase coordinates) are least stable; the circles are all high on the Y axis in Figure 10. There are four points in Figure 10 that contradict the trend, one high-energy *C*_1_ structure and three low-energy *C*_i_ structures.

One point in Figure 10 that contradicts the hypothesis that lattice-free *C*_i_ dimers are least stable is the red diamond corresponding to **8b**, an unstable *C*_1_ dimer. However, this molecule has the most aromatic groups and is the densest non-*C*_i_ dimer. The four aromatic groups in **8b** interact extensively which is readily apparent upon examination of the packing. The method of the calculations summarized by Figure 10 replaced the aromatic substituents with hydrogen atoms. The difference between **8a** and **8b** is *i*Pr versus Tol at the amidine N atoms (R^3^). Perhaps surface area-dependent dispersive interactions in the nucleation process of C_1_ dimer, **8b** perturbed hydrogen bonding away from optimum. Aromatic stacking is quoted anywhere between 2 and 0.5 kcal/mol so a scenario in which the eight aromatic-interactions in dimer **8b** perturbed the energies of the hydrogen bonds is very reasonable.

The red circles in Figure 10 represent *C*_i_ dimer **9b1**–**9b3** cocrystallized 1:1 with EtOAc, Toluene, and Et_2_O respectively. The three red out-of-place circles to the left of the graph in Figure 8 also belong to **9b1**–**9b3**. Molecule **9b** was the only one in this study to crystallize with solvent. Further attempts to obtain **9b** solvent-free resulted in 1:1 inclusions of CH_3_CN, isopropyl ether, and chlorobenzene which were not analyzed completely. Solvent appears in ~15% of neutral organics in the CSD and has been attributed to interrupted crystallization processes [38]. The presence of solvent molecules in **9b** neatly allows rationalization of the high densities of **9b1**–**9b3** and the low, per molecule, gas-phase energies, and the low-energy θ parameters. From these observations we make the following hypothesis. *The solution states of* ***9b*** *did not completely desolvate during the nucleation process thereby maintaining near solution-state conformation. This allowed the low calculated energies, and allowed the low-energy θ parameters. Disordered solvent molecules in the crystalline phase of* ***9*** *occupied voids at the faces of the two hydrogen-bound imidazole rings thereby increasing the densities of* ***9*** *and preserving near solution-state conformation for the C_i_ dimer of ****9***.

**Figure 11:**
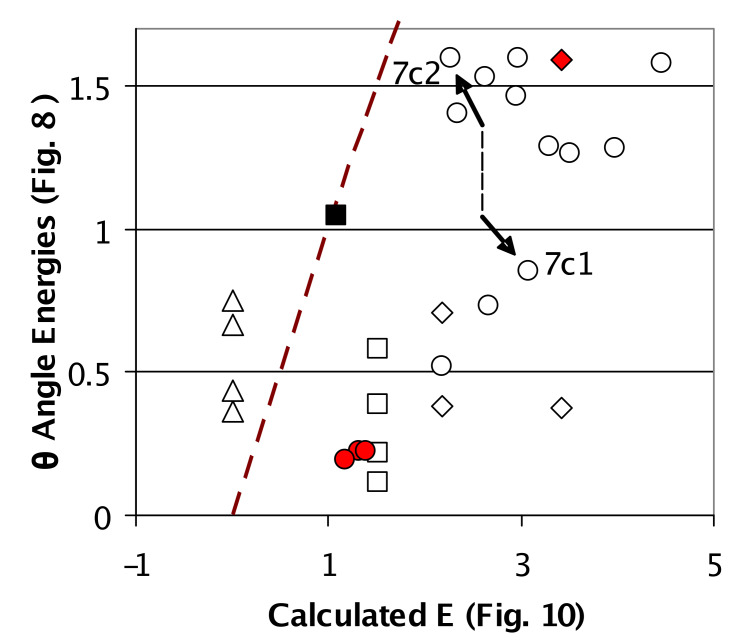
The icon legend is identical to Figure 8 and Figure 10. The Y-axis from Figure 8 energies (θ only) and the X-axis from Figure 10 energies are compared. The high and low energies of θ are mostly responsible for the two crystalline phases that lie outside the prediction that the gas-phase *C*_i_ dimer should be high-energy. Dashed line has slope = 1.

More significance of calculations of θ and of the hydrogen bond motifs was attained by plotting the energies in Figure 10 against the energies in Figure 8. If all the points in Figure 11 had fallen on a diagonal line, the energies in Figure 10 would have been ascribable to the θ parameter of Figure 8 with no motif-dependent, energetic differences from intermolecular hydrogen bonding. However, Figure 11 shows that tetramer **9a** (triangles) optimized hydrogen bonds better (smaller X axis values) than trimer **6b** (open squares) even though the tetramer had less optimum θ parameters (larger average Y axis values) than the trimer. The sign of θ alternates around the tetrameric ring, thus point symmetry possibilities for the reduction of Z' in this structure were *C*_2_ and *S*_4_, but not *C*_i_. Axiomatically, wide XH-Y angles stabilize hydrogen bonds [39]; crystal structures prefer ~linear hydrogen bonds like those in Figure 6 for tetramer **9a**, over the less linear hydrogen bonds in **5c** and **11a** in Figure 5 and Figure 7 [14–16].

Figure 11 also supports the hypothesis that inter-dimer dispersive forces edited the optimum solution state conformation and n-mer molecularity. The low-energy *C*_i_ dimers **9b1**–**3** (red circles in Figure 11) corresponded to the *C*_i_ dimers with the lowest-energy θ angles in Figure 8. Little perturbation of the solution state occurred upon nucleation because EtOAc, toluene and ether solvent molecules filled the voids and preserved the optimum solution-state conformation. In each case, the lacuna in the lattices occupied by solvent allows for much disorder on the part of the solvent. These three points really do not at all contradict the hypothesis that bringing *C*_i_ symmetric dimers of **2** from solution into the solid state results in low-energy packing and high-energy local interactions.

The density of the crystalline phase of **7c** decreased in going from truly *C*_i_ symmetric **7c1** to near *C*_i_ symmetric **7c2** in Figure 10. Figure 11 analyzes this change in terms of hydrogen bonding and θ-derived energies. Attaining *C*_i_ symmetry is accompanied by stabilization of θ-derived energies (~0.74 kcal/mol) and destabilization of hydrogen bonding (0.80 kcal/mol). Within error these effects all but cancel and this result is consonant with the view that polymorphism is the result of a subtle balance of orthogonal forces associated with certain atomic parameters [40]. A relatively large increase in density accompanied the attainment of true *C*_i_ symmetry. The caveat here is of course that only one polymorph was found and thus generalizations will have to wait for a study of another system.

The *C*_i_ dimers in general had less stable hydrogen bonds and non-optimum θ angles which put them in the upper right corner of the graph in Figure 11. The *C*_1_ dimer also suffers due to high-energy hydrogen bonds (right side of Figure 11) but this dimer can optimize one of the two structures in the asymmetric unit as in **8b**, or both fairly well as in **8a**, thereby minimizing the average local interaction energy. The high-energy molecular component in *C*_1_ dimer **8b** (high-energy red diamond in Figure 11) corresponded to the highest-energy θ angle in Figure 8 that lost π resonance between the amidine and the imidazole moiety. As discussed earlier, this structure is likely a result of extensive π-stacking.

## Conclusion

In a family of molecules this study found 15 crystalline phases in which the hydrogen bonded motif was *C*_i_ symmetric, Z' = 1 and five other structures in which the components of the hydrogen bonded motif were not related by improper symmetry. Other than the tape motif in which the hydrogen bonded components followed a screw axis, the other four structures had Z' ≥ 2. The packing of *C*_i_ symmetric pairs stabilized this most popular motif even though the *C*_i_ motif was destabilized relative to other motifs by conformational and hydrogen bonding effects. Our analysis of this small data set separated local versus dispersive contributions to stability. In Gavezzotti's statistical search of the CSD for energy-edited symmetry preferences in Z' = 2 vs. Z' = 1 structures, a relationship was found between solid state symmetry and the stabilities of pair-wise interactions [26]. In related work, Steed *et al.* found that stereogenic atoms included in normally *C*_i_ symmetric hydrogen bonded dimers, increases Z' from 1 to 2, indicating that rotational symmetry is less propitious in the minimization of Z' than inversion [41]. Very related to the current report is Wheeler's discovery that heterochiral isosteric molecules conserve the solid state inversion-symmetric motifs of their racemic analogues [42–43]. These results relate to Wallach's hypothesis: either packing interactions are optimal when *C*_i_-symmetric units nucleate or *C*_i_-symmetric interactions are more stable in solution and hence get included in the crystalline phase. Regarding less condensed states, optically pure gas phase methyl lactate favors the tetramer over dimers more than the racemic mixture [44]. The lack of the energetically competitive heterochiral dimer in the optically pure mixture could have produced that result.

With all things equal, apparently packing prefers centro-symmetric pairs. Here, structures other than the *C*_i_ dimer required more stability from hydrogen bonding and conformation to compensate for non-optimal packing forces. Here, less dense crystalline phases resulted when packing forces yielded control of conformation and hydrogen bond motif to local, more directional forces.

Should all molecules capable of hydrogen bonding build lattices of *C*_i_ symmetric aggregates with Z' = 1 at the expense of local interactions? No, local and dispersive forces can also synergize to construct the solid state. Crystal structure databases are likely mosaics containing molecular families with structural aspects that compete and synergize to varying degrees. More work has to be put toward a holistic understanding of interplay between packing and solid state synthons that are usually the function of strong local interactions [45]. However when there is competition between local interactions and the dispersive interactions, this work suggests that small *C*_i_ symmetric units have a slight thermodynamic packing advantage which could be the basis for Wallach's rule.

The results bring into question predictive methods based on energy minimization and their level of accuracy, especially in the prediction of hydrogen bonding options that are proximal in energy [28,46–47]. Prediction of the organic solid state is challenged by the fact that many crystalline phases likely result from marginal differences in large opposing effects. While the problem of calculating marginal differences in large energetic contributions to the organic solid state has been the subject of previous conjecture, this study is one example of the nature of the problem, unveiled and dissected.

The results provide a caveat for mining crystal structure databases and translating structural popularity to energy. Such searches should be as structurally broad as possible. For example, tendencies of a particular dihedral angle to adopt a certain average value could be gleaned by looking at many crystal structures. However the applicability of the parameter outside the solid state may be severely and systematically diminished by symmetry-edited packing effects. The most common dihedral angle is the highest-energy dihedral angle from Figure 8 with a fairly large data set. This study detailed relevant interactions in how such an observation could occur. Similar statements can be made about the most popular hydrogen bond motif followed by caveats regarding the use of crystal-structure derived atomic parameters to broadly characterize hydrogen bonding energies.

## Supporting Information

File 1 Experimental Section. The Experimental Section describes the synthesis and purification of all substances described, and general experimental procedures.

File 2 Crystal Data and Structure Refinement Information

File 3 Sample ^1^H NMR Spectra for **10b**, and **14b**

File 4 Crystallographic Information Files (CIF) for all the compounds reported in this work. File Names: Xy_descriptor.cif. Xy is the compound number and letter designation found in Table I and/or described in the Experimental Section. The descriptor is a short tag indicating hydrogen bond motif or symmetry.
