# Supplementary material for: Inversion symmetry and local vs. dispersive interactions in the nucleation of hydrogen bonded cyclic n-mer and tape of imidazolecarboxamidines
Source: Beilstein J Org Chem. 2008 Jul 7;4:23. doi: 10.3762/bjoc.4.23 (PMC2511023; doi:10.3762/bjoc.4.23)
Supplement: File 1 — Experimental Section. The Experimental Section describes the synthesis and purification of all substances described, and general experimental procedures. [file Beilstein_J_Org_Chem-04-23-s001.pdf]

Supporting Information File 1

Experimental Section

Inversion symmetry and local vs. dispersive interactions in the nucleation of hydrogen bonded cyclic n-mer and tape of imidazolecarboxamidines

*Sihui Long, Venkatraj Muthusamy, Peter G. Willis, Sean Parkin and Arthur Cammers**

Address: University of Kentucky, Department of Chemistry, Lexington, KY. 40506-0055

Inversion Symmetry and Local vs. Dispersive Interactions in the Nucleation of Hydrogen Bonded Cyclic n-mer and Tape of Imidazolecarboxamidines

Experimental Section

General. Melting points are uncorrected. IR spectra were recorded using FT-IR Nicolet 560 spectrometer. NMR spectra were obtained on a Varian Gemini and Varian INOVA spectrometers at ^{1}H observation frequencies 200 and 400 MHz. NMR spectra for characterization purposes taken in $CDCl_3$ were complicated due to broadening by aggregation and slow exchange of amidine rotamers; DMSO-d_6 clarified some of these. Solvent-dependent kinetics were previously investigated [1].

N,N'-Ditolyl-4,5-dimethylimidazole-1-carboxamidine **5b.** A mixture of 4,5-dimethylimidazole (85 mg, 0.88 mmol) and *N,N'*-di-tolylcarbodiimide (210 mg, 0.94 mmol) was refluxed in 10 mL of THF for 12 h. THF was evaporated and the residues were fractionated by flash chromatography: EtOAc (R_f = 0.42), to afford a white solid. Suitable crystals were obtained from EtOAc via slow evaporation. 200 mg (71%); mp 150–153 °C; IR (cm^{-1}) 1662, 1607, 1553, 1505; ^{1}H NMR (DMSO-d_6, 400 MHz) δ 9.62 (bs, 1H), 7.64 (bd, J = 8.0 Hz, 2H), 7.52 (s, 1H), 7.12 (d, J = 8.0 Hz, 2H), 6.92 (d, J = 8.0 Hz, 2H), 6.50 (d, J = 8.0 Hz, 2H), 2.26 (s, 3H), 2.18 (s, 3H), 2.00 (s, 3H), 1.96 (s, 3H); ^{13}C NMR (DMSO-d_6, 100 MHz): δ 145.1, 139.0, 137.6, 135.1, 132.7, 132.0, 131.0, 129.8 (br. exchange), 124.3, 122.4, 122.0, 119.9, 20.9 (br. exchange), 12.6, 9.1; MS (EI) *m/z* 317([M-H]$^{+}$); $C_{20}H_{22}N_4$ (318)

Analogous methods afforded the compounds below.

N,N'-Dicyclohexyl-4,5-dimethylimidazole-1-carboxamidine **5c**. Flash chromatography: EtOAc (R_f = 0.35). 200 mg (53%) of a white solid. Crystals were obtained by slow evaporation in EtOAc. mp 122–125 °C; IR (cm^{-1}) 3254.0, 2925.7, 2851.5, 1666.7, 1580.6, 1524.3; ^{1}H NMR ($CDCl_3$, 400 MHz) δ7.306 (s, 1H), 2.169 (s, 3H), 2.081 (s, 3H), 2.0–1.5 (m, 10H), 1.5-1.0 (m, 10H); ^{13}C NMR ($CDCl_3$, 100 MHz) δ 133.577, 133.372, 121.394, 34.971, 33.931, 25.855, 25.088, 24.974, 24.777, 12.737, 8.502; MS (EI) m/z 302 (M+); $C_{18}H_{30}N_4$ (302).

N,N'-Ditolyl-2-methylimidazole-1-carboxamidine **6b.** Flash chromatography: EtOAc (R_f = 0.38), to afford 150 mg (81%) of a white solid. Crystals were obtained by slow evaporation of EtOAc. mp 138–141 °C; IR (cm^{-1}) 1655, 1625, 1556, 1532; ^{1}H NMR (400 MHz, $CDCl_3$) δ 7.1–6.8 (m, 10H), 6.5 (bs, 1H) 2.28 (s, 3H), 2.20 (bs, 6H); ^{13}C NMR (100 MHz, $CDCl_3$) δ 130.3, 130.0 128.1, 124.1, 120.8, 119.0, 21.2, 14.3; MS (EI) *m*/*z* 304 (M^+); $C_{19}H_{20}N_4$ (304).

N,N'-Dicyclohexyl-2-methylimidazole-1-carboxamidine **6c**. Flash chromatography: EtOAc, to afford 300 mg (50%) of a white solid. Crystals were obtained by slow evaporation in EtOAc. mp 140–142 °C; IR (cm^{-1}) 1672, 1531; ^{1}H NMR (400 MHz, $CDCl_3$) δ 6.90 (bs, 1H), 6.79 (bs, 1H), 4.1–3.0 (bm, 3H), 2.30 (s, 3H), 1.8–1.0 (m, 10H); ^{13}C NMR (100 MHz, $CDCl_3$) δ 143.9, 140.0 br, 128.0, 118.3, 58-50 br, 25.8, 24.8, 13.1; MS (EI) *m*/*z* 288 (M^+); $C_{17}H_{28}N_4$ (288).

N,N'-Diisopropyl-2,4,5-trimethylimidazole-1-carboxamidine **7a**. Flash chromatography EtOAc (R_f = 0.1), to afford 100 mg (52%) of a white solid. Crystals were obtained by slow evaporation in hexane. mp 120–123 °C; IR (cm^{-1}) 1659, 1533; ^{1}H NMR ($CDCl_3$,

400 MHz) δ 3.8 (bs, 1H), 2.9 (s, 1H), 2.28 (s, 3H), 2.10 (s, 3H), 2.04 (s, 3H), 1.11 (br, 10H); ^{13}C NMR ($CDCl_3$, 100 MHz) δ 141.3, 132.4, 121.1, 13.3, 12.5, 8.8; MS (EI) *m*/*z* 236 (M^+); $C_{13}H_{24}N_4$ (236).

N,N'-Ditolyl-2,4,5-trimethylimidazole-1-carboxamidine **7b**. Flash chromatography EtOAc (R_f = 0.28), to afford 87 mg (72%) of a white solid. Crystals were obtained by slow evaporation in EtOAc. mp 176–178 °C; IR (cm^{-1}) 1659, 1606, 1553; ^{1}H NMR ($CDCl_3$, 400 MHz) δ 8.6–6.2 (br, 8H), 2.28 (br, 6H), 2.13 (br, 3H), 2.07 (br, 3H), 2.00 (br, 3H); ^{13}C NMR ($CDCl_3$, 100 MHz) δ133.0 br, 129.7 br, 121.6 (br), 119.5 (br), 29.9, 21.0, 13.9, 12.4, 9.5; MS (EI) *m*/*z* 332 (M^+); $C_{21}H_{24}N_4$ (332).

N,N'-Dicyclohexyl-2,4,5-trimethylimidazole-1-carboxamidine **7c**. Flash chromatography, eluting with EtOAc (R_f = 0.15), to afford 380 mg (88%) of a white solid. Crystals were obtained by slow evaporation of EtOAc/Hexane. mp 132–134 °C; IR (cm^{-1}) 1664.08, 1525.84; ^{1}H NMR ($CDCl_3$, 400 MHz) δ 3.85 (s, 1H), 2.95 (bs, 2H), 2.25 (s, 3H), 2.09 (s, 3H), 2.02 (s, 3H), 1.8–1.4 (m, 10H), 1.4-1.0 (br, 10H); ^{13}C NMR ($CDCl_3$, 100 MHz) δ 141.3, 139.5, 132.2, 121.1, 56.9 br, 50.0 br, 35.1 br, 34.9 br, 25.8, 25.5 br, 24.8, 13.3, 12.6, 8.9 MS (EI) *m*/*z* 316 (M^+); $C_{19}H_{32}N_4$ (316).

N,N'-Diisopropyl-4,5-diphenylimidazole-1-carboxamidine **8a**. Upon cooling much 4,5-diphenylimidazole precipitated from solution. Flash chromatography: EtOAc (R_f = 0.49), 80 mg (29%) as a white solid. Crystals were grown in EtOAc. mp 169–171 °C; IR (cm^{-1}) 1673, 1618, 1577, 1526; (NMR DMSO-d_6: multiple species present) ^{1}H NMR ($CDCl_3$, 400 MHz) δ 7.56 (s, 1H), 7.51–7.16 (m, 10H), 3.8–3.0 (bm 2H), 1.24–0.90 (bm, 10H);

^{13}C NMR (100 MHz, $CDCl_3$) δ 138.1, 135.9, 134.1, 129.9, 128.8, 128.7, 128.4, 127.5, 127.1, 29.9, 22.8 br; MS (EI) *m*/*z* 346 (M^+); $C_{22}H_{26}N_4$ (346).

N,N'-Ditolyl-4,5-diphenylimidazole-1-carboxamidine **8b**. Flash chromatography: CH_2Cl_2 forerun followed by 10:1 CH_2Cl_2: EtOAc, to afford 120 mg (36%) of a white solid. Crystals were obtained by slow evaporation in EtOAc. mp 142–145 °C; IR (cm^{-1}) 1658.82, 1604.23, 1546.45, 1504.90; ^1H NMR (400 MHz, $CDCl_3$) δ 8.1–6.7 (br, m, 19H), 6.05 (br, 1H), 2.23 (br, 6H); ^{13}C NMR (100 MHz, $CDCl_3$) δ 136.8, 130.3, 130.2, 129.6, 129.2, 129.0, 129.0, 128.6, 128.4, 128.3, 127.7, 127.5, 127.2, 120.9, 21.0; MS (EI) *m*/*z* 442(M^+); $C_{30}H_{26}N_4$ (442).

N,N'-Diisopropylimidazole-1-carboxamidine **9a**. Imidazole (0.07 g, 1 mmol) and *N,N'*-diisopropylcarbodiimide (0.18 g, 1.2 mmol) were refluxed in THF (4 mL) for 12 h. Upon cooling, crystals of **9a** formed and were collected by filtration. Recrystallization from hexane afforded colorless crystals (0.14 g, 72 %); mp 75–77 ˚C; IR (cm^{-1}) 1672, 1559; ^1H NMR (400 MHz, $CDCl_3$) δ 7.85 (br s, 1H), 7.58 (br s, 2H), 7.22 (br s, 1H), 7.11 (br s, 2H), 7.06 (br s, 1H), 7.00 (br s, 2H), 3.2–3.8 (m, 9H), 1.2–1.1 (br s, 36 H); ^{13}C NMR (100 MHz, $CDCl_3$) δ; 144.2, 139.0, 136.5, 129.5, 118.3, 48.9 (b), 43.7 (b), 24.0 (b); MS (EI) *m*/*z* 194(M^+); $C_{10}H_{18}N_4$ (194.28).

Analogous methods afforded the compounds below.

N,N'-Ditolylimidazole-1-carboxamidine **9b**. Diffraction-quality crystals were obtained upon cooling in EtOAc, toluene, ether, CH_3CN, iso-propyl ether, or cholorbenzene. (0.22 g, 76 %); mp 122–124 ˚C; IR (cm^{-1}) 1664, 1555; ^1H NMR (400 MHz, $CDCl_3$) δ 7.90 (br s, 1H), 7.03 (m, 8H), 6.66 (br s, 2H), 6.22 (br s, 1H), 2.28 (br s, 6H); ^{13}C NMR (100

MHz, $CDCl_3$) δ; 137.0, 130.2, 129.8, 129.6, 124.1, 121.4, 118.3, 21.2; MS (EI) *m*/*z* 290(M^+); $C_{18}H_{18}N_4$ (290.37).

N,N'-Dicyclohexylimidazole-1-carboxamidine **9c**. Diffraction-quality crystals were obtained upon cooling in EtOAc. (0.19 g, 70 %); mp 130–132 ˚C; IR (cm^{-1}) 3275, 1675, 1530, 1487; ^{1}H NMR (400 MHz, $CDCl_3$) δ 7.60 (br s, 2H), 7.00 (br s, 1H), 3.10 (br s, 2H), 1.9–1.0 (m, 21H); ^{13}C NMR (100 MHz, $CDCl_3$) δ; 136, 129, 118, 34.6, 33.8, 25.8, 25.6, 24.9, 24.6; MS (EI) *m*/*z* 274(M^+); $C_{16}H_{26}N_4$ (274.41).

N,N'-Ditolylbenzimidazole-1-carboxamidine **10b**. Flash chromatography: 6:1 CH_2Cl_2/EtOAc, to afford 270 mg (78%) of a white solid. Crystals were obtained by slow evaporation in EtOAc. mp 159–160 °C; IR (cm^{-1}) 1665.88, 1606.21, 1549.98; ^{1}H NMR ($CDCl_3$, 400 MHz) δ 8.2-6.4 (m, 13H), 2.27 (br, 6H); ^{1}H NMR (DMSO-d_6, 400 MHz) δ 9.87 (s, 1H), 8.29 (s, 1H), 7.62–7.67 (m, 2H), 7.40 (d, J = 7.1, 1H), 7.19–7.26 (m, 3H), 7.16 (d, J = 7.4, 2H), 6.82 (d, J = 7.1, 2H), 6.52 (d, J = 7.4, 2H), 2.28 (3H), 2.09 (3H); ^{13}C NMR ($CDCl_3$, 100 MHz) δ 142.1, 130.4, 124.8, 123.9, 121.1, 120.6, 21.0; ^{13}C NMR (DMSO-d_6, 100 MHz) δ 144.8, 142.8, 142.1, 137.9, 137.3, 132.4, 131.8, 131.1, 130.3, 129.1, 123.8, 123.6, 122.6, 121.0, 119.6, 119.3, 111.6, 20.5, 20.3 (methyl signals exchange at 25°C); MS (EI) *m*/*z* 340 (M^+); $C_{22}H_{20}N_4$ (340).

N,N'-Dicyclohexylbenzimidazole-1-carboxamidine **10c**. Flash chromatography: EtOAc (R_f = 0.5), 285 mg (86%) of a white solid. Crystals were obtained by slow evaporation in EtOAc. mp 114–117 °C; IR (cm^{-1}) 1665.33, 1613.42, 1529.56; ^{1}H NMR ($CDCl_3$, 400 MHz) δ 8.2–7.3 (m, br, 5H), 4.09 (br, 1H), 3.22 (br, 1H), 2.90 (br, 1H), 1.90–1.00 (m, 20H); The chemical shifts and coupling in the ABMX portion of the following spectrum

were extracted via simulation, gNMR v. 4.1.0, 1999. ^{1}H NMR (DMSO-d_6, 400 MHz) δ 8.26 (s, 1H), 7.72 (d, J = 7.7 Hz, 1H), 7.37 (d, J = 7.8 Hz, 1H), 7.31 (dd, J = 7.7, 7.3 Hz, 1H), 7.26 (dd, J = 7.7, 7.4 Hz, 1H), 6.68 (d, J = 7.1 Hz, 1H, NH), 2.07–1.95 (m, 2H), 1.79–1.66 (m, 4H), 1.62–1.52 (m, 4H), 1.49–1.35 (m, 4H), 1.32–1.14 (m, 8H); ^{13}C NMR (DMSO-d_6, 100 MHz) δ 156.6, 142.2, 138.5, 133.1, 123.4, 122.2, 119.6, 110.7, 56.1, 49.8, 35.1, 31.7, 25.6, 25.2, 24.5, 24.1; MS (EI) *m/z* 324 (M^+); $C_{20}H_{28}N_4$ (324).

N,N'-Diisopropyl-2-methylbenzimidazole-1-carboxamidine, **11a.** Crystals were obtained by slow evaporation in EtOAc. (0.96 g, 74 %); mp 124–126 ˚C; IR (cm^{-1}) 1657, 1537; ^{1}H NMR (400 MHz, $CDCl_3$) δ 7.70 (d, J = 2.8 Hz, 1H), 7.26 (br s, 3H), 3.99 (br s, 1H), 2.87 (br s, 1H), 2.57 (s, 3H), 1.3-1.1 (br s, 12H); ^{13}C NMR (100 MHz, $CDCl_3$) δ; 149.8, 142.1, 138.5, 134.2, 122.9, 122.5, 119.0, 109.7, 48.7(b), 43.5(b), 24.5(b), 13.5; MS (EI) *m/z* 258(M^+); $C_{15}H_{22}N_4$ (258.37).

N,N'-Ditolyl-2-methylbenzimidazole-1-carboxamidine **11b**. Flash chromatography: EtOAc, 250 mg (70%) of a white solid. Crystals were obtained by slow evaporation in EtOAc. mp 199–200 °C; IR (cm^{-1}) 1651, 1604, 1546; The chemical shifts and coupling in the ABMX portion of the following spectrum were extracted via simulation. ^{1}H NMR (DMSO-d_6, 400 MHz) δ 9.83 (s, 1H), 7.73–7.67 (m, 2H), 7.51 (dd, J = 8.0, 1.9 Hz, 1H), 7.38 (dd, J = 8.0, 1.9 Hz, 1H), 7.18 (dd, J = 8.1, 7.4 Hz, 1H), 7.16 (dd, J = 8.0, 7.4 Hz, 1H), 7.19–7.14 (m, 2H), 6.81 (d, J = 8.0 Hz, 2H), 6.49 (d, J = 8.0 Hz, 2H), 2.40 (s, 3H), 2.28 (s, 3H), 2.08 (s, 3H); ^{13}C NMR (100 MHz, DMSO-d_6) δ 149.9, 144.4, 141.8, 137.9, 137.3, 134.5, 131.9, 131.4, 129.2, 129.1, 122.8, 122.2, 121.0, 119.2, 118.5, 110.6, 20.5, 20.2, 14.2; MS (EI) *m/z* 354(M^+); $C_{23}H_{22}N_4$ (354).

N,N'-Dicyclohexyl-2-methylbenzimidazole-1-carboxamidine **11c**. Flash chromatography: EtOAc (R_f = 0.5), 235 mg (77%) of a white solid. Crystals were obtained by slow evaporation in EtOAc. mp 152–155 °C; IR (cm^{-1}) 1663.02, 1529.85; ^{1}H NMR (DMSO-d_6, 400 MHz) δ 7.57 (dm, *J* = 6.2 Hz, 1H), 7.23-7.16 (m, 2H), 7.21 (s, 1H), 6.65 (d, *J* = 7.51 Hz, 1H), 3.64 (s, 1H), 2.42 (s, 3H), 2.40–2.33 (m, 1H), 2.07–1.94 (m, 2H), 1.78–1.64 (m, 2H) , 1.64–1.48 (m, 2H), 1.47–0.72 (m, 14H); ^{13}C NMR (100 MHz, $CDCl_3$) δ150.2, 142.5, 134.5, 123.2, 122.8, 119.3, 110.8, 110.0, 97.2 (br), 52.8, 50.5, 35.1 (br), 34.2, 33.9, 33.0 (br), 25.9, 25.7, 25.3, 25.2, 24.8, 13.9; ^{13}C NMR (100 MHz, DMSO-d_6) δ 149.8, 141.9, 138.6, 134.5, 122.4, 121.8, 118.4, 109.7, 56.1, 49.6, 35.0, 34.8, 31.7, 31.5, 25.6, 25.2, 24.5, 24.1, 13.4; MS (EI) *m/z* 338(M^+); $C_{21}H_{30}N_4$(338).

The following compounds were not included in the argument due to the involvement of the amino group at the 2-position of the imidazole moiety in hydrodrogen bonding. The focus of this work was a solid state involving imidazole as a hydrogen bond acceptor and amidine as a hydrogen bond donor. In one case, the 2-amino group participated in the chemistry of the addition, giving rise to an unexpected product.

2-Amino-N,N'-Diisopropylbenzimidazole-1-carboxamidine **12a**. A mixture of 2-amino-benzimidazole (1.11 g, 8.31 mmol) and the N,N'-diisopropylcarbodiimide (3.7 g, 16.6 mmol) was dissolved in THF (20 mL) and the solution was refluxed for 12 h. Crude product was extracted from water into $CHCl_3$. Recrystallization of the residue from ethyl acetate afforded colorless crystals (1.82 g, 85 %); IR (cm^{-1}) 1770, 1554; ^{1}H NMR (300 MHz, $CDCl_3$) δ; 7.40 (d, 1H), 7.27 (br d, 1H), 7.14 (t, 1H), 7.05 (t, 1H), 5.2–6.0 (br s, 2H), 3.2–4.2 (br m, 4H), 1.0–1.4 (br s, 12H); ^{13}C NMR (75.4 MHz, $CDCl_3$) δ; 160.6, 153,

141.9, 133.1, 122.6, 116.7, 109.7 (b), 47.0 (b), 24.1 (b); Analysis calculated for $C_{14}H_{21}N_5$ (259.35): C 64.83; H 8.16; N 27.01; found: C 64.51; H 8.31; N 26.55.

2-Amino-N,N'-diisopropyl-5,6-dimethylbenzimidazole-1-carboxamidine **13a.** mp 195–197 ˚C; IR (cm^{-1}) 1651, 1552; ^{1}H NMR (300 MHz, $CDCl_3$) δ; 7.17 (s, 1H), 7.03 (s, 1H), 5.36 (br s, 2H), 3.8–3.4 (m, 2H), 2.31 (s, 6H), 1.25–1.13 (br s, 12H); ^{13}C NMR (300 MHz, $CDCl_3$) δ; 163.7, 152.5, 140.0, 131.0, 129.0, 117.3, 110.5 (vb), 47.2 (vb), 24.2 (b), 20.5; MS (EI) m/z 287(M^+); $C_{16}H_{25}N_5$ (287.4).

7,8-Dimethyl-2-tolylamino-4-(tolylimino)-3-tolyl-[1,3,5]triazino[1,2-a]benzimidazole **14b.** (Figure 12) Flash chromatography: EtOAc 350 mg (87%) of a white solid. Crystals were obtained by slow evaporation in EtOAc. mp 163–165 °C; IR (cm^{-1}) 3398, 1686, 1632, 1598(s), 1561, 1528, 1509; ^{1}H NMR (400 MHz, $CDCl_3$) δ 8.00 (s, 1H), 7.50 (s, 1H), (Tol. *o*-cplg. analyzed as doublets) 7.36 (d, 2H, J = 9.0 Hz), 7.06 (d, 2H, J = 9.0 Hz), 7.05 (d, 2H, J = 8.4 Hz), 7.00 (d, 2H, J = 8.4 Hz), 6.72 (d, 2H, J = 8.0 Hz), 6.31 (d, 2H, J = 8.0 Hz), 6.13 (br, 1H), 2.38 (s, 3H), 2.32 (s, 3H), 2.31 (s, 3H), 2.28 (s, 3H), 2.21 (s, 3H); ^{13}C NMR (100 MHz, CDCl3) δ 150.1, 149.6, 143.3, 141.0, 141.7 (br), 134.7, 134.4, 133.4, 132.0, 131.7, 131.1, 131.0, 130.6, 130.5, 129.6, 128.8, 128.4, 121.4, 120.8, 118.6, 115.9, 21.3, 21.0, 20.8, 20.6, 20.5; MS (EI) m/z 499(M+); $C_{32}H_{30}N_6$(calc. 499.2); 500(M+1, rel. int. 37.4%, calc. 37.2%); 501(M+2, rel. int. 7.1%, calc. 6.8%).

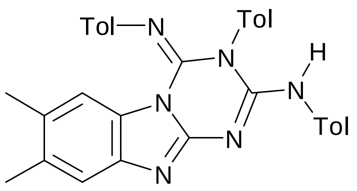

Figure 12. Anomalous product, **14b**.

Crystal structure determinations. Data on all the compounds were collected with a Nonius kappaCCD diffractometer; cell refinement and data reduction were done using SCALEPACK and DENZO-SMN [2]. Structure solution and refinement were carried out using the SHELXS97 and SHELXL97 program, respectively [3]. Parameters in CIF format are available as electronic supplementary information.

References:

1. Willis, P.G. Designing Molecular Recognition in the Context of Hydrogen Bonding and Molecular Dynamics. Dissertation, University of Kentucky, Lexington, KY, 2001, pp. 1-40.

2. Otwinowski, Z.; Minor, W. in *Processing of x-ray diffraction data collected in oscillation mode*, *Vol. 276;* Carter, C. W. J.; Sweet, R. M., Eds.; Academic Press: San Diego, CA, 1997, pp. 307-326.

3. Sheldrick, G. M. SHELX97 - Programs for Crystal Structure Solution and Refinement, University of Göttingen, Germany, 1997.
