# Supplementary material for: Inversion symmetry and local vs. dispersive interactions in the nucleation of hydrogen bonded cyclic n-mer and tape of imidazolecarboxamidines
Source: Beilstein J Org Chem. 2008 Jul 7;4:23. doi: 10.3762/bjoc.4.23 (PMC2511023; doi:10.3762/bjoc.4.23)
Supplement: File 2 — Crystal Data and Structure Refinement Information [file Beilstein_J_Org_Chem-04-23-s002.pdf]

Supporting Information File 2

Crystal Data and Structure Refinement Information

Inversion symmetry and local vs. dispersive interactions in the nucleation of hydrogen bonded cyclic n-mer and tape of imidazolecarboxamidines

*Sihui Long, Venkatraj Muthusamy, Peter G. Willis, Sean Parkin and Arthur Cammers**

Address: University of Kentucky, Department of Chemistry, Lexington, KY. 40506-0055

Table 2. Crystal Data and Structure Refinement Information.

Compound 5b

Empirical formula	C20 H22 N4
Formula weight	318.42
Temperature	90.0(2) K
Wavelength	0.71073 A
Crystal system, space group	Triclinic, P -1
Unit cell dimensions	a = 9.32870(10) A alpha = 87.4755(6) deg.<br>b = 9.47900(10) A beta = 70.5173(6) deg.<br>c = 10.4921(2) A gamma = 76.4271(6) deg.
Volume	849.64(2) A^3
Z, Calculated density	2, 1.245 Mg/m^3
Absorption coefficient	0.076 mm^-1
F(000)	340
Crystal size	0.20 x 0.20 x 0.08 mm
Theta range for data collection	2.06 to 27.47 deg.
Limiting indices	-12<=h<=12, -12<=k<=12, -13<=l<=13
Reflections collected / unique	7568 / 3876 [R(int) = 0.0217]
Completeness to theta = 27.47	99.8 %
Absorption correction	Semi-empirical from equivalents
Max. and min. transmission	0.9943 and 0.9850
Refinement method	Full-matrix least-squares on F^2
Data / restraints / parameters	3876 / 0 / 221
Goodness-of-fit on F^2	1.058
Final R indices [I>2sigma(I)]	R1 = 0.0421, wR2 = 0.1057
R indices (all data)	R1 = 0.0538, wR2 = 0.1137
Largest diff. peak and hole	.229 and -.269 e.A^-3

Compound 5c

Empirical formula	C18 H30 N4
Formula weight	302.46
Temperature	90.0(2) K
Wavelength	0.71073 A
Crystal system, space group	Monoclinic, P 21/c
Unit cell dimensions	

a = 14.3412(3) A alpha = 90 deg.
b = 10.9327(3) A beta = 91.0293(11) deg.
c = 11.1408(4) A gamma = 90 deg.

Volume	1746.46(9) A^3
Z, Calculated density	4, 1.150 Mg/m^3
Absorption coefficient	0.070 mm^-1
F(000)	664
Crystal size	0.26 x 0.25 x 0.15 mm
Theta range for data collection	1.42 to 27.48 deg.
Limiting indices	-18<=h<=18, -12<=k<=14, -14<=l<=14
Reflections collected / unique	7383 / 4010 [R(int) = 0.0334]
Completeness to theta = 27.48	99.9 %
Absorption correction	Semi-empirical from equivalents
Max. and min. transmission	0.9896 and 0.9821
Refinement method	Full-matrix least-squares on F^2
Data / restraints / parameters	4010 / 0 / 202
Goodness-of-fit on F^2	1.070
Final R indices [I>2sigma(I)]	R1 = 0.0443, wR2 = 0.1077
R indices (all data)	R1 = 0.0740, wR2 = 0.1196
Extinction coefficient	0.0076(18)
Largest diff. peak and hole	.193 and -.226 e.A^-3

Compound 6b

Empirical formula	C19 H20 N4
Formula weight	304.39
Temperature	90.0(2) K
Wavelength	0.71073 A
Crystal system, space group	Monoclinic, P 21/c
Unit cell dimensions	

a = 17.0057(2) A alpha = 90 deg.
b = 8.9486(1) A beta = 98.2202(6) deg.
c = 34.3882(5) A gamma = 90 deg.

Volume	5179.33(11) A^3
Z, Calculated density	12, 1.171 Mg/m^3
Absorption coefficient	0.072 mm^-1
F(000)	1944
Crystal size	0.30 x 0.28 x 0.25 mm
Theta range for data collection	1.20 to 24.00 deg.
Limiting indices	-19<=h<=19, -10<=k<=10, -39<=l<=39
Reflections collected / unique	15636 / 8132 [R(int) = 0.0525]
Completeness to theta = 24.00	100.0 %
Absorption correction	Semi-empirical from equivalents
Max. and min. transmission	0.9823 and 0.9788
Refinement method	Full-matrix least-squares on F^2
Data / restraints / parameters	8132 / 560 / 765
Goodness-of-fit on F^2	1.051
Final R indices [I>2sigma(I)]	R1 = 0.0670, wR2 = 0.1751
R indices (all data)	R1 = 0.1268, wR2 = 0.2107
Largest diff. peak and hole	.757 and -.416 e.A^-3

Compound 6c

Empirical formula	C17 H28 N4
Formula weight	288.43
Temperature	90.0(2) K
Wavelength	0.71073 A
Crystal system, space group	Monoclinic, C 2/c

Unit cell dimensions
a = 33.1028(5) A alpha = 90 deg.
b = 6.16430(10) A beta = 125.5810(11) deg.
c = 20.2723(4) A gamma = 90 deg.

Volume	3364.33(10) A^3
Z, Calculated density	8, 1.139 Mg/m^3
Absorption coefficient	0.069 mm^-1
F(000)	1264
Crystal size	0.40 x 0.30 x 0.15 mm
Theta range for data collection	1.51 to 25.00 deg.
Limiting indices	-37<=h<=38, -7<=k<=7, -24<=l<=23
Reflections collected / unique	5633 / 2968 [R(int) = 0.0392]
Completeness to theta = 25.00	100.0 %
Absorption correction	None
Refinement method	Full-matrix least-squares on F^2
Data / restraints / parameters	2968 / 0 / 191
Goodness-of-fit on F^2	1.028
Final R indices [I>2sigma(I)]	R1 = 0.0516, wR2 = 0.1242
R indices (all data)	R1 = 0.0890, wR2 = 0.1436
Largest diff. peak and hole	.245 and -.224 e.A^-3

Compound 7a

Empirical formula	C13 H24 N4
Formula weight	236.36
Temperature	90.0(2) K
Wavelength	0.71073 A
Crystal system, space group	Triclinic, P -1
Unit cell dimensions	

a = 8.5317(2) A alpha = 81.8531(12) deg.
b = 8.7993(2) A beta = 68.8269(12) deg.
c = 11.5447(4) A gamma = 61.5111(14) deg.

Volume	709.83(3) A^3
Z, Calculated density	2, 1.106 Mg/m^3
Absorption coefficient	0.068 mm^-1
F(000)	260
Crystal size	0.40 x 0.30 x 0.15 mm
Theta range for data collection	1.89 to 27.40 deg.
Limiting indices	-11<=h<=11, -11<=k<=11, -14<=l<=14
Reflections collected / unique	6280 / 3229 [R(int) = 0.0371]
Completeness to theta = 27.40	99.8 %
Absorption correction	Semi-empirical from equivalents
Max. and min. transmission	0.9898 and 0.9731
Refinement method	Full-matrix least-squares on F^2
Data / restraints / parameters	3229 / 0 / 161
Goodness-of-fit on F^2	1.046
Final R indices [I>2sigma(I)]	R1 = 0.0497, wR2 = 0.1262
R indices (all data)	R1 = 0.0830, wR2 = 0.1443
Largest diff. peak and hole	.233 and -.255 e.A^-3

Compound 7b

Empirical formula	C21 H24 N4
Formula weight	332.44
Temperature	90.0(2) K
Wavelength	0.71073 A
Crystal system, space group	Monoclinic, P 21/n
Unit cell dimensions	

a = 9.8002(1) A   alpha = 90 deg.
b = 7.2445(1) A   beta = 94.6725(6) deg.
c = 26.1183(4) A   gamma = 90 deg.

Volume	1848.17(4) A^3
Z, Calculated density	4, 1.195 Mg/m^3
Absorption coefficient	0.072 mm^-1
F(000)	712
Crystal size	0.20 x 0.14 x 0.08 mm
Theta range for data collection	1.56 to 27.48 deg.
Limiting indices	-12<=h<=12, -9<=k<=9, -33<=l<=33
Reflections collected / unique	8154 / 4245 [R(int) = 0.0289]
Completeness to theta = 27.48	100.0 %
Absorption correction	Semi-empirical from equivalents
Max. and min. transmission	0.9942 and 0.9857
Refinement method	Full-matrix least-squares on F^2
Data / restraints / parameters	4245 / 0 / 231
Goodness-of-fit on F^2	1.058
Final R indices [I>2sigma(I)]	R1 = 0.0460, wR2 = 0.1194
R indices (all data)	R1 = 0.0729, wR2 = 0.1342
Largest diff. peak and hole	.255 and -.273 e.A^-3

Compound 7c1 Ci

Empirical formula	C19 H32 N4
Formula weight	316.49
Temperature	90.0(2) K
Wavelength	1.54178 A
Crystal system, space group	Monoclinic, P 21/n
Unit cell dimensions	

a = 9.3059(6) A alpha = 90 deg.
b = 12.1138(8) A beta = 100.910(2) deg.
c = 16.5226(11) A gamma = 90 deg.

Volume	1828.9(2) A^3
Z, Calculated density	4, 1.149 Mg/m^3
Absorption coefficient	0.530 mm^-1
F(000)	696
Crystal size	0.12 x 0.10 x 0.08 mm
Theta range for data collection	4.56 to 69.38 deg.
Limiting indices	-11<=h<=11, -14<=k<=11, -20<=l<=19
Reflections collected / unique	26867 / 3408 [R(int) = 0.0394]
Completeness to theta = 69.38	99.4 %
Absorption correction	Semi-empirical from equivalents
Max. and min. transmission	0.959 and 0.859
Refinement method	Full-matrix least-squares on F^2
Data / restraints / parameters	3408 / 0 / 212
Goodness-of-fit on F^2	1.031
Final R indices [I>2sigma(I)]	R1 = 0.0357, wR2 = 0.0896
R indices (all data)	R1 = 0.0379, wR2 = 0.0919
Extinction coefficient	0.0021(3)
Largest diff. peak and hole	0.215 and -0.175 e.A^-3

Compound 7c2 C1

Empirical formula	C19 H32 N4
Formula weight	316.49
Temperature	150.0(2) K
Wavelength	0.71073 A
Crystal system, space group	Monoclinic, P 21/c

Unit cell dimensions

a = 11.5364(2) A alpha = 90 deg.
b = 10.5199(2) A beta = 97.9299(7) deg.
c = 31.4132(6) A gamma = 90 deg.

Volume	3775.91(12) A^3
Z, Calculated density	8, 1.113 Mg/m^3
Absorption coefficient	0.067 mm^-1
F(000)	1392
Crystal size	0.28 x 0.25 x 0.25 mm
Theta range for data collection	1.31 to 25.00 deg.
Limiting indices	-13<=h<=13, -12<=k<=12, -37<=l<=37
Reflections collected / unique	12797 / 6644 [R(int) = 0.0525]
Completeness to theta = 25.00	99.9 %
Absorption correction	Semi-empirical from equivalents
Max. and min. transmission	0.9834 and 0.9814
Refinement method	Full-matrix least-squares on F^2
Data / restraints / parameters	6644 / 0 / 421
Goodness-of-fit on F^2	0.988
Final R indices [I>2sigma(I)]	R1 = 0.0521, wR2 = 0.1281
R indices (all data)	R1 = 0.1119, wR2 = 0.1549
Largest diff. peak and hole	.206 and -.187 e.A^-3

Compound 8a

Empirical formula	C22 H26 N4
Formula weight	346.47
Temperature	90.0(2) K
Wavelength	0.71073 A
Crystal system, space group	Triclinic, P -1

Unit cell dimensions
a = 10.7318(2) A alpha = 106.3484(7) deg.
b = 11.9553(2) A beta = 91.1499(7) deg.
c = 17.3861(3) A gamma = 111.0528(7) deg.

Volume	1979.23(6) A^3
Z, Calculated density	4, 1.163 Mg/m^3
Absorption coefficient	0.070 mm^-1
F(000)	744
Crystal size	0.20 x 0.20 x 0.15 mm
Theta range for data collection	1.23 to 27.48 deg.
Limiting indices	-13<=h<=13, -15<=k<=15, -22<=l<=22
Reflections collected / unique	18009 / 9081 [R(int) = 0.0631]
Completeness to theta = 27.48	99.9 %
Absorption correction	None
Refinement method	Full-matrix least-squares on F^2
Data / restraints / parameters	9081 / 0 / 478
Goodness-of-fit on F^2	0.971
Final R indices [I>2sigma(I)]	R1 = 0.0527, wR2 = 0.1149
R indices (all data)	R1 = 0.1196, wR2 = 0.1394
Extinction coefficient	0.0052(10)
Largest diff. peak and hole	.426 and -.445 e.A^-3

Compound 8b

Empirical formula	C30 H26 N4
Formula weight	442.55
Temperature	90.0(2) K
Wavelength	0.71073 A
Crystal system, space group	Monoclinic, P 21/n
Unit cell dimensions	
a = 11.94010(10) A alpha = 90 deg.	
b = 16.43130(10) A beta = 95.0436(4) deg.	
c = 25.0618(2) A gamma = 90 deg.	
Volume	4897.87(6) A^3
Z, Calculated density	8, 1.200 Mg/m^3
Absorption coefficient	0.072 mm^-1
F(000)	1872
Crystal size	0.20 x 0.15 x 0.13 mm
Theta range for data collection	1.48 to 27.49 deg.
Limiting indices	-15<=h<=15, -21<=k<=21, -32<=l<=32
Reflections collected / unique	22087 / 11245 [R(int) = 0.0455]
Completeness to theta = 27.49	100.0 %
Absorption correction	Semi-empirical from equivalents
Max. and min. transmission	0.9907 and 0.9858
Refinement method	Full-matrix least-squares on F^2
Data / restraints / parameters	11245 / 0 / 618
Goodness-of-fit on F^2	1.005
Final R indices [I>2sigma(I)]	R1 = 0.0464, wR2 = 0.1080
R indices (all data)	R1 = 0.0932, wR2 = 0.1260
Extinction coefficient	0.0010(3)
Largest diff. peak and hole	.218 and -.242 e.A^-3

Compound 9a

Empirical formula	C10 H18 N4
Formula weight	194.28
Temperature	90.0(2) K
Wavelength	0.71073 A
Crystal system, space group	Monoclinic, P 21/n
Unit cell dimensions	

a = 15.6729(2) A alpha = 90 deg.
b = 14.6136(2) A beta = 103.6249(5) deg.
c = 21.7183(3) A gamma = 90 deg.

Volume	4834.32(11) A^3
Z, Calculated density	16, 1.068 Mg/m^3
Absorption coefficient	0.068 mm^-1
F(000)	1696
Crystal size	0.30 x 0.12 x 0.10 mm
Theta range for data collection	1.45 to 25.00 deg.
Limiting indices	-18<=h<=18, -17<=k<=17, -25<=l<=25
Reflections collected / unique	16655 / 8520 [R(int) = 0.0558]
Completeness to theta = 25.00	100.0 %
Absorption correction	Semi-empirical from equivalents
Max. and min. transmission	0.9933 and 0.9800
Refinement method	Full-matrix least-squares on F^2
Data / restraints / parameters	8520 / 0 / 533
Goodness-of-fit on F^2	0.988
Final R indices [I>2sigma(I)]	R1 = 0.0499, wR2 = 0.1077
R indices (all data)	R1 = 0.1065, wR2 = 0.1272
Largest diff. peak and hole	.273 and -.219 e.A^-3

Compound 9b1

Empirical formula	C20 H22 N4 O
Formula weight	334.42
Temperature	90.0(2) K
Wavelength	0.71073 A
Crystal system, space group	Monoclinic, P 21/n

Unit cell dimensions
a = 5.7171(1) A alpha = 90 deg.
b = 13.9901(3) A beta = 93.1226(8) deg.
c = 22.3180(5) A gamma = 90 deg.

Volume	1782.41(6) A^3
Z, Calculated density	4, 1.246 Mg/m^3
Absorption coefficient	0.080 mm^-1
F(000)	712
Crystal size	0.30 x 0.30 x 0.30 mm
Theta range for data collection	1.72 to 25.00 deg.
Limiting indices	-6<=h<=6, -16<=k<=16, -26<=l<=26
Reflections collected / unique	27124 / 3139 [R(int) = 0.0483]
Completeness to theta = 25.00	99.9 %
Absorption correction	Semi-empirical from equivalents
Max. and min. transmission	0.9765 and 0.9765
Refinement method	Full-matrix least-squares on F^2
Data / restraints / parameters	3139 / 81 / 256
Goodness-of-fit on F^2	1.024
Final R indices [I>2sigma(I)]	R1 = 0.0570, wR2 = 0.1599
R indices (all data)	R1 = 0.0813, wR2 = 0.1781
Largest diff. peak and hole	0.608 and -0.435 e.A^-3

Compound 9b2

Empirical formula	C21.50 H22 N4
Formula weight	336.43
Temperature	90.0(2) K
Wavelength	0.71073 A
Crystal system, space group	Monoclinic, P 21/n
Unit cell dimensions	

a = 5.7467(1) A alpha = 90 deg.
b = 13.8294(3) A beta = 93.377(1) deg.
c = 22.1701(5) A gamma = 90 deg.

Volume	1758.87(6) A^3
Z, Calculated density	4, 1.270 Mg/m^3
Absorption coefficient	0.077 mm^-1
F(000)	716
Crystal size	0.5 x 0.1 x 0.1 mm
Theta range for data collection	1.74 to 27.48 deg.
Limiting indices	-7<=h<=7, -17<=k<=17, -28<=l<=28
Reflections collected / unique	26115 / 4043 [R(int) = 0.0485]
Completeness to theta = 27.48	100.0 %
Absorption correction	Semi-empirical from equivalents
Max. and min. transmission	0.992 and 0.962
Refinement method	Full-matrix least-squares on F^2
Data / restraints / parameters	4043 / 394 / 327
Goodness-of-fit on F^2	1.044
Final R indices [I>2sigma(I)]	R1 = 0.0694, wR2 = 0.1947
R indices (all data)	R1 = 0.1201, wR2 = 0.2269
Largest diff. peak and hole	0.355 and -0.873 e.A^-3

Compound 9b3

Empirical formula	C20 H23 N4 O0.50
Formula weight	327.42
Temperature	90.0(2) K
Wavelength	0.71073 A
Crystal system, space group	Monoclinic, P 21/n
Unit cell dimensions	

a = 5.7147(1) A alpha = 90 deg.
b = 13.9252(2) A beta = 93.0935(7) deg.
c = 22.2681(3) A gamma = 90 deg.

Volume	1769.48(5) A^3
Z, Calculated density	4, 1.229 Mg/m^3
Absorption coefficient	0.077 mm^-1
F(000)	700
Crystal size	0.40 x 0.25 x 0.20 mm
Theta range for data collection	1.83 to 27.49 deg.
Limiting indices	-7<=h<=7, -18<=k<=17, -28<=l<=28
Reflections collected / unique	23516 / 4064 [R(int) = 0.0261]
Completeness to theta = 27.49	99.8 %
Absorption correction	Semi-empirical from equivalents
Max. and min. transmission	0.985 and 0.970
Refinement method	Full-matrix least-squares on F^2
Data / restraints / parameters	4064 / 71 / 248
Goodness-of-fit on F^2	1.083
Final R indices [I>2sigma(I)]	R1 = 0.0617, wR2 = 0.1722
R indices (all data)	R1 = 0.0849, wR2 = 0.1904
Largest diff. peak and hole	0.653 and -0.722 e.A^-3

Compound 10b

Empirical formula	C22 H20 N4
Formula weight	340.42
Temperature	90.0(2) K
Wavelength	1.54178 A
Crystal system, space group	Triclinic, P -1

Unit cell dimensions
a = 5.7578(3) A alpha = 86.213(2) deg.
b = 10.3819(4) A beta = 79.332(3) deg.
c = 15.1645(7) A gamma = 84.967(2) deg.

Volume	886.24(7) A^3
Z, Calculated density	2, 1.276 Mg/m^3
Absorption coefficient	0.605 mm^-1
F(000)	360
Crystal size	0.12 x 0.02 x 0.01 mm
Theta range for data collection	2.97 to 67.96 deg.
Limiting indices	-6<=h<=5, -12<=k<=12, -18<=l<=18
Reflections collected / unique	11087 / 3138 [R(int) = 0.0560]
Completeness to theta = 67.96	97.3 %
Absorption correction	Semi-empirical from equivalents
Max. and min. transmission	0.9952 and 0.8923
Refinement method	Full-matrix least-squares on F^2
Data / restraints / parameters	3138 / 0 / 238
Goodness-of-fit on F^2	1.035
Final R indices [I>2sigma(I)]	R1 = 0.0444, wR2 = 0.1076
R indices (all data)	R1 = 0.0643, wR2 = 0.1177
Extinction coefficient	0.0027(7)
Largest diff. peak and hole	.272 and -.206 e.A^-3

Compound 10c

Empirical formula	C20 H28 N4
Formula weight	324.46
Temperature	90.0(2) K
Wavelength	0.71073 A
Crystal system, space group	Triclinic, P -1
Unit cell dimensions	

a = 8.9442(5) A alpha = 74.610(3) deg.
b = 9.2587(6) A beta = 82.151(3) deg.
c = 12.0545(8) A gamma = 68.098(3) deg.

Volume	892.20(10) A^3
Z, Calculated density	2, 1.208 Mg/m^3
Absorption coefficient	0.073 mm^-1
F(000)	352
Crystal size	0.30 x 0.12 x 0.08 mm
Theta range for data collection	1.75 to 27.44 deg.
Limiting indices	-11<=h<=11, -11<=k<=11, -15<=l<=15
Reflections collected / unique	7629 / 4039 [R(int) = 0.0451]
Completeness to theta = 27.44	99.3 %
Absorption correction	Semi-empirical from equivalents
Max. and min. transmission	0.9942 and 0.9784
Refinement method	Full-matrix least-squares on F^2
Data / restraints / parameters	4039 / 0 / 217
Goodness-of-fit on F^2	1.048
Final R indices [I>2sigma(I)]	R1 = 0.0576, wR2 = 0.1345
R indices (all data)	R1 = 0.1011, wR2 = 0.1551
Largest diff. peak and hole	.286 and -.370 e.A^-3

Compound 11a

Empirical formula	C15 H22 N4
Formula weight	258.37
Temperature	90.0(2) K
Wavelength	0.71073 A
Crystal system, space group	Monoclinic, P 21/c
Unit cell dimensions	

a = 8.7820(3) A alpha = 90 deg.
b = 7.9720(2) A beta = 94.7240(13) deg.
c = 21.1910(7) A gamma = 90 deg.

Volume	1478.54(8) A^3
Z, Calculated density	4, 1.161 Mg/m^3
Absorption coefficient	0.072 mm^-1
F(000)	560
Crystal size	0.20 x 0.20 x 0.20 mm
Theta range for data collection	1.93 to 27.49 deg.
Limiting indices	-11<=h<=11, -10<=k<=10, -27<=l<=27
Reflections collected / unique	6456 / 3393 [R(int) = 0.0563]
Completeness to theta = 27.49	99.9 %
Absorption correction	None
Refinement method	Full-matrix least-squares on F^2
Data / restraints / parameters	3393 / 0 / 177
Goodness-of-fit on F^2	0.968
Final R indices [I>2sigma(I)]	R1 = 0.0516, wR2 = 0.1124
R indices (all data)	R1 = 0.1120, wR2 = 0.1369
Largest diff. peak and hole	.209 and -.267 e.A^-3

Compound 11b

Empirical formula	C23 H22 N4
Formula weight	354.45
Temperature	90.0(2) K
Wavelength	0.71073 A
Crystal system, space group	Monoclinic, P 21/n

Unit cell dimensions
a = 7.22820(10) A alpha = 90 deg.
b = 14.5797(3) A beta = 94.6029(8) deg.
c = 18.0219(4) A gamma = 90 deg.

Volume	1893.11(6) A^3
Z, Calculated density	4, 1.244 Mg/m^3
Absorption coefficient	0.075 mm^-1
F(000)	752
Crystal size	0.20 x 0.20 x 0.15 mm
Theta range for data collection	1.80 to 27.47 deg.
Limiting indices	-9<=h<=9, -18<=k<=18, -23<=l<=23
Reflections collected / unique	8482 / 4332 [R(int) = 0.0350]
Completeness to theta = 27.47	100.0 %
Absorption correction	Semi-empirical from equivalents
Max. and min. transmission	0.9888 and 0.9851
Refinement method	Full-matrix least-squares on F^2
Data / restraints / parameters	4332 / 0 / 247
Goodness-of-fit on F^2	1.037
Final R indices [I>2sigma(I)]	R1 = 0.0483, wR2 = 0.1195
R indices (all data)	R1 = 0.0825, wR2 = 0.1367
Largest diff. peak and hole	.267 and -.279 e.A^-3

Compound 11c

Empirical formula	C21 H30 N4
Formula weight	338.49
Temperature	90.0(2) K
Wavelength	0.71073 A
Crystal system, space group	Triclinic, P -1
Unit cell dimensions	

a = 9.3343(4) A alpha = 70.786(2) deg.
b = 9.6107(4) A beta = 74.417(2) deg.
c = 12.4635(7) A gamma = 67.211(2) deg.

Volume	960.51(8) A^3
Z, Calculated density	2, 1.170 Mg/m^3
Absorption coefficient	0.071 mm^-1
F(000)	368
Crystal size	0.40 x 0.25 x 0.08 mm
Theta range for data collection	1.75 to 25.00 deg.
Limiting indices	-10<=h<=11, -10<=k<=11, 0<=l<=14
Reflections collected / unique	3378 / 3378 [R(int) = 0.0848]
Completeness to theta = 25.00	100.0 %
Absorption correction	Semi-empirical from equivalents
Max. and min. transmission	0.9944 and 0.9723
Refinement method	Full-matrix least-squares on F^2
Data / restraints / parameters	3378 / 0 / 227
Goodness-of-fit on F^2	1.046
Final R indices [I>2sigma(I)]	R1 = 0.0476, wR2 = 0.1158
R indices (all data)	R1 = 0.0698, wR2 = 0.1299
Largest diff. peak and hole	.217 and -.232 e.A^-3

Compound 12a

Empirical formula	C18 H29 N5 O2
Formula weight	347.46
Temperature	173(1) K
Wavelength	0.71073 A
Crystal system, space group	Orthorhombic, P na21
Unit cell dimensions	

a = 18.066(4) A alpha = 90 deg.
b = 10.234(2) A beta = 90 deg.
c = 22.275(5) A gamma = 90 deg.

Volume	4118.4(14) A^3
Z, Calculated density	8, 1.121 Mg/m^3
Absorption coefficient	0.075 mm^-1
F(000)	1504
Crystal size	0.44 x 0.30 x 0.30 mm
Theta range for data collection	1.83 to 22.50 deg.
Limiting indices	-19<=h<=19, -11<=k<=11, -23<=l<=23
Reflections collected / unique	19888 / 5371 [R(int) = 0.0762]
Completeness to theta = 22.50	99.9 %
Absorption correction	None
Refinement method	Full-matrix least-squares on F^2
Data / restraints / parameters	5371 / 417 / 451
Goodness-of-fit on F^2	1.116
Final R indices [I>2sigma(I)]	R1 = 0.0737, wR2 = 0.1638
R indices (all data)	R1 = 0.0908, wR2 = 0.1728
Absolute structure parameter	0.00
Largest diff. peak and hole	.228 and -.239 e.A^-3

Compound 13a

Empirical formula	C16 H25 N5
Formula weight	287.41
Temperature	173(1) K
Wavelength	0.71073 A
Crystal system, space group	Monoclinic, P 21/n
Unit cell dimensions	
a = 8.0430(2) A alpha = 90 deg.	
b = 25.6630(9) A beta = 113.7590(18)	deg.
c = 8.5490(2) A gamma = 90 deg.	
Volume	1615.03(8) A^3
Z, Calculated density	4, 1.182 Mg/m^3
Absorption coefficient	0.074 mm^-1
F(000)	624
Crystal size	0.35 x 0.32 x 0.20 mm
Theta range for data collection	2.72 to 27.54 deg.
Limiting indices	-10<=h<=10, -32<=k<=33, -11<=l<=11
Reflections collected / unique	11294 / 3694 [R(int) = 0.0398]
Completeness to theta = 27.54	99.2 %
Absorption correction	Semi-empirical from equivalents
Max. and min. transmission	0.9854 and 0.9747
Refinement method	Full-matrix least-squares on F^2
Data / restraints / parameters	3694 / 0 / 197
Goodness-of-fit on F^2	1.030
Final R indices [I>2sigma(I)]	R1 = 0.0583, wR2 = 0.1246
R indices (all data)	R1 = 0.0796, wR2 = 0.1339
Extinction coefficient	0.011(3)
Largest diff. peak and hole	.507 and -.414 e.A^-3

Compound 14b

Empirical formula	C32 H30 N6
Formula weight	498.62
Temperature	90.0(2) K
Wavelength	0.71073 A
Crystal system, space group	Triclinic, P -1

Unit cell dimensions
a = 10.2471(2) A alpha = 73.0483(8) deg.
b = 10.8387(2) A beta = 69.9698(9) deg.
c = 13.1028(2) A gamma = 88.6849(7) deg.

Volume	1303.04(4) A^3
Z, Calculated density	2, 1.271 Mg/m^3
Absorption coefficient	0.077 mm^-1
F(000)	528
Crystal size	0.40 x 0.20 x 0.10 mm
Theta range for data collection	1.74 to 27.49 deg.
Limiting indices	-13<=h<=13, -13<=k<=14, -16<=l<=17
Reflections collected / unique	11646 / 5975 [R(int) = 0.0426]
Completeness to theta = 27.49	99.9 %
Absorption correction	Semi-empirical from equivalents
Max. and min. transmission	0.9923 and 0.9697
Refinement method	Full-matrix least-squares on F^2
Data / restraints / parameters	5975 / 0 / 348
Goodness-of-fit on F^2	1.002
Final R indices [I>2sigma(I)]	R1 = 0.0485, wR2 = 0.1162
R indices (all data)	R1 = 0.0932, wR2 = 0.1353
Largest diff. peak and hole	.269 and -.254 e.A^-3
