# Supplementary material for: Inversion symmetry and local vs. dispersive interactions in the nucleation of hydrogen bonded cyclic n-mer and tape of imidazolecarboxamidines
Source: Beilstein J Org Chem. 2008 Jul 7;4:23. doi: 10.3762/bjoc.4.23 (PMC2511023; doi:10.3762/bjoc.4.23)
Supplement: File 3 — Sample 1H NMR Spectra for 10b, and 14b [file Beilstein_J_Org_Chem-04-23-s003.pdf]

Inversion Symmetry and Local vs. Dispersive Interactions in the Nucleation of Hydrogen Bonded Cyclic n-mer and Tape of Imidazolecarboxamidines

Supplementary Information, Sample 1H NMR Spectra, Compound **10b**, and **14b**.

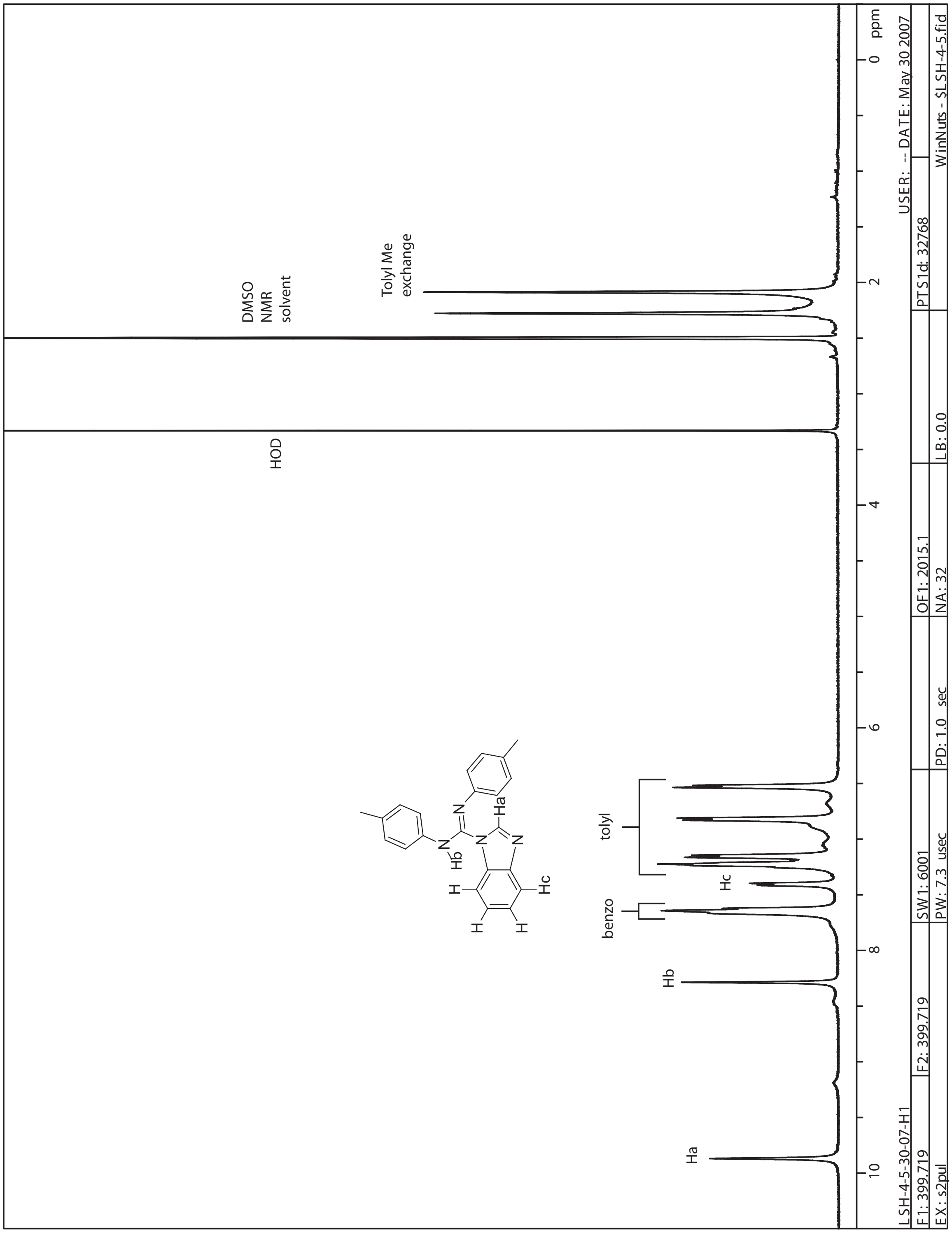

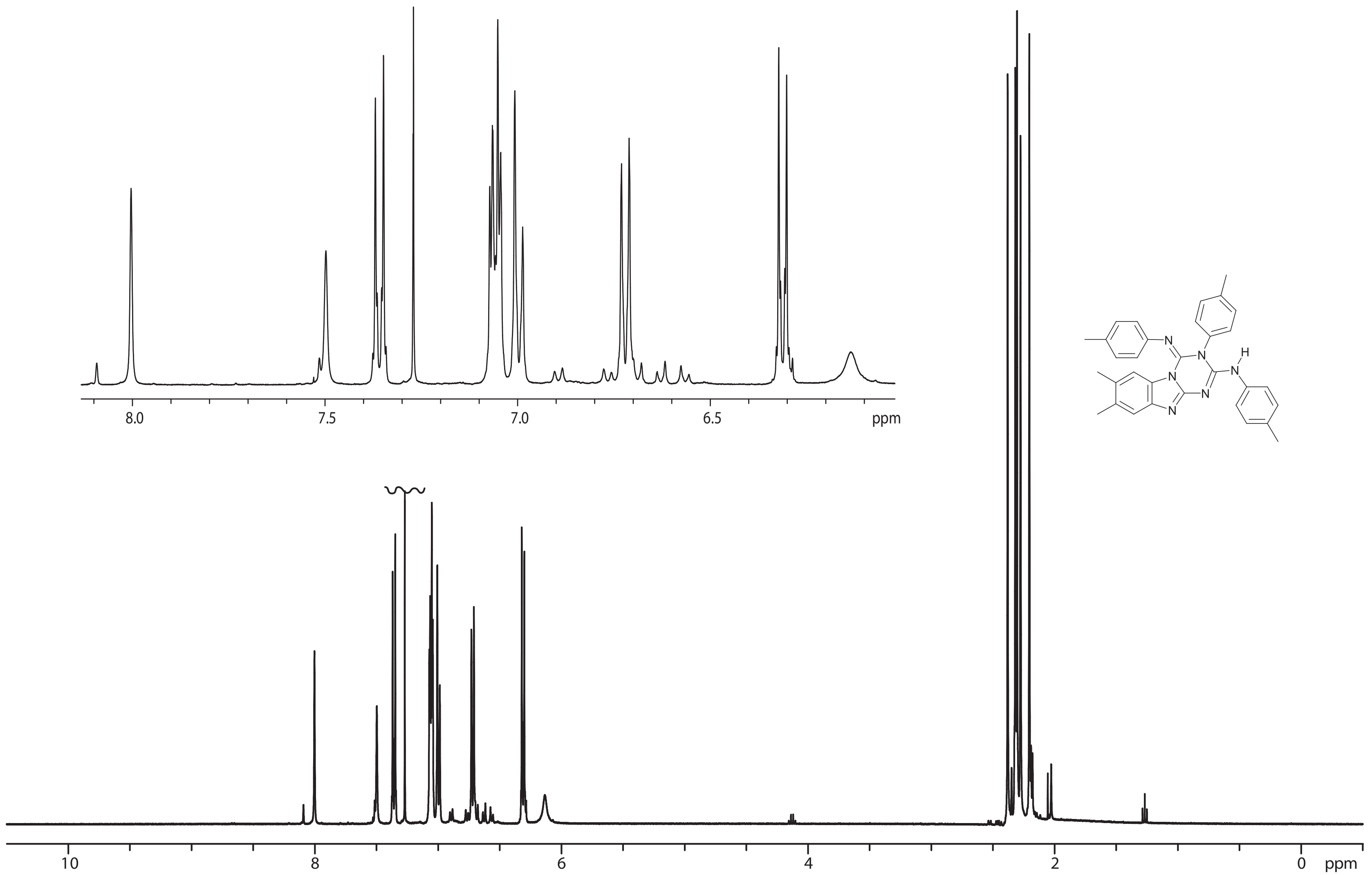

LSH-38-10-05							USER: -- DATE: Aug 10 2005
F1: 399.723	F2: 399.723	SW1: 6001		OF1: 2008.0		PTS1d: 32768	
EX: s2pul		PW: 7.3 usec	PD: 1.0 sec	NA: 64	LB: 0.0		WinNuts - $14a
